# Human CD56^+^CD39^+^ dNK cells support fetal survival through controlling trophoblastic cell fate: immune mechanisms of recurrent early pregnancy loss

**DOI:** 10.1093/nsr/nwae142

**Published:** 2024-04-11

**Authors:** Wentong Jia, Liyang Ma, Xin Yu, Feiyang Wang, Qian Yang, Xiaoye Wang, Mengjie Fan, Yan Gu, Ran Meng, Jian Wang, Yuxia Li, Rong Li, Xuan Shao, Yan-Ling Wang

**Affiliations:** State Key Laboratory of Stem cell and Reproductive Biology, Key Laboratory of Organ Regeneration and Reconstruction, Institute of Zoology, Chinese Academy of Sciences, Beijing 100101, China; State Key Laboratory of Stem cell and Reproductive Biology, Key Laboratory of Organ Regeneration and Reconstruction, Institute of Zoology, Chinese Academy of Sciences, Beijing 100101, China; State Key Laboratory of Stem cell and Reproductive Biology, Key Laboratory of Organ Regeneration and Reconstruction, Institute of Zoology, Chinese Academy of Sciences, Beijing 100101, China; Beijing Institute for Stem Cell and Regenerative Medicine, Beijing 100101, China; University of the Chinese Academy of Sciences, Beijing 101408, China; State Key Laboratory of Stem cell and Reproductive Biology, Key Laboratory of Organ Regeneration and Reconstruction, Institute of Zoology, Chinese Academy of Sciences, Beijing 100101, China; Beijing Institute for Stem Cell and Regenerative Medicine, Beijing 100101, China; University of the Chinese Academy of Sciences, Beijing 101408, China; NHC Key Lab of Reproduction Regulation, Shanghai Engineering Research Center of Reproductive Health Drug and Devices, Shanghai Institute for Biomedical and Pharmaceutical Technologies, Shanghai 200237, China; National Clinical Center for Obstetrics and Gynecology, Peking University Third Hospital, Beijing 100191, China; National Clinical Center for Obstetrics and Gynecology, Peking University Third Hospital, Beijing 100191, China; Department of Family Planning, The Second Hospital of Tianjin Medical University, Tianjin 300211, China; Department of Prenatal Screening, Haidian Maternal and Child Health Hospital, Beijing 100080, China; NHC Key Lab of Reproduction Regulation, Shanghai Engineering Research Center of Reproductive Health Drug and Devices, Shanghai Institute for Biomedical and Pharmaceutical Technologies, Shanghai 200237, China; State Key Laboratory of Stem cell and Reproductive Biology, Key Laboratory of Organ Regeneration and Reconstruction, Institute of Zoology, Chinese Academy of Sciences, Beijing 100101, China; National Clinical Center for Obstetrics and Gynecology, Peking University Third Hospital, Beijing 100191, China; State Key Laboratory of Stem cell and Reproductive Biology, Key Laboratory of Organ Regeneration and Reconstruction, Institute of Zoology, Chinese Academy of Sciences, Beijing 100101, China; Beijing Institute for Stem Cell and Regenerative Medicine, Beijing 100101, China; University of the Chinese Academy of Sciences, Beijing 101408, China; State Key Laboratory of Stem cell and Reproductive Biology, Key Laboratory of Organ Regeneration and Reconstruction, Institute of Zoology, Chinese Academy of Sciences, Beijing 100101, China; Beijing Institute for Stem Cell and Regenerative Medicine, Beijing 100101, China; University of the Chinese Academy of Sciences, Beijing 101408, China

**Keywords:** recurrent pregnancy loss, CD56^+^CD39^+^ dNK subset, NOG mice, adoptive transfer, placental trophoblast cell fate, M-CSF

## Abstract

Decidual natural killer (dNK) cells are the most abundant immune cells at the maternal-fetal interface during early pregnancy in both mice and humans, and emerging single-cell transcriptomic studies have uncovered various human dNK subsets that are disrupted in patients experiencing recurrent early pregnancy loss (RPL) at early gestational stage, suggesting a connection between abnormal proportions or characteristics of dNK subsets and RPL pathogenesis. However, the functional mechanisms underlying this association remain unclear. Here, we established a mouse model by adoptively transferring human dNK cells into pregnant NOG (NOD/Shi-*scid*/IL-2Rγ^null^) mice, where human dNK cells predominantly homed into the uteri of recipients. Using this model, we observed a strong correlation between the properties of human dNK cells and pregnancy outcome. The transfer of dNK cells from RPL patients (dNK-RPL) remarkably worsened early pregnancy loss and impaired placental trophoblast cell differentiation in the recipients. These adverse effects were effectively reversed by transferring CD56^+^CD39^+^ dNK cells. Mechanistic studies revealed that CD56^+^CD39^+^ dNK subset facilitates early differentiation of mouse trophoblast stem cells (mTSCs) towards both invasive and syncytial pathways through secreting macrophage colony-stimulating factor (M-CSF). Administration of recombinant M-CSF to NOG mice transferred with dNK-RPL efficiently rescued the exacerbated pregnancy outcomes and fetal/placental development. Collectively, this study established a novel humanized mouse model featuring functional human dNK cells homing into the uteri of recipients and uncovered the pivotal role of M-CSF in fetal-supporting function of CD56^+^CD39^+^ dNK cells during early pregnancy, highlighting that M-CSF may be a previously unappreciated therapeutic target for intervening RPL.

## INTRODUCTION

The establishment of an appropriate immune-adaptative microenvironment at the maternal-fetal interface is crucial for successful pregnancy in viviparous mammals, relying primarily on the intricate and dynamic interactions between placental trophoblast cells and various maternal immune cells in the uterine decidua [1]. Aberrant immune responses in the decidua have been shown to disrupt embryonic and placental development, resulting in a range of pregnancy disorders, such as recurrent pregnant loss (RPL), typically characterized by three or more consecutive spontaneous pregnancy losses before 24 weeks gestational age [2–4].

Decidual natural killer (dNK) cells represent the predominant population of immune cells at the maternal-fetal interface during early gestation, constituting ∼70% of uterine leukocytes [5]. Unlike CD3^−^CD56^dim^CD16^+^ peripheral NK (pNK) cells, human CD3^−^CD56^bright^CD16^−^ dNK cells exhibit lower cytotoxicity but higher capacity in producing immunomodulatory cytokines and growth factors [6,7], which play essential roles in modulating uterine immune adaptation and fetal-placental development [8–10]. Importantly, human dNK cells enhance invasion of placental extravillous trophoblast (EVT) cells and remodeling of uterine spiral arteries, through direct ligand-receptor recognition between trophoblast-specific major histocompatibility complex (MHC) molecules and receptors expressed by dNK cells such as killer cell immunoglobulin-like receptors (KIRs), or indirect interactions mediated by cytokines secreted by dNK cells including GM-CSF, PIGF, VEGF-C, HGF, etc. [11–14].

Recently, studies utilizing single cell RNA-sequencing (scRNA-seq) analysis have identified distinct molecular characteristics of several subsets of human dNK cells [15–17]. Among these subsets, dNK1 cells specifically express *CD39* and exhibit high levels of *KIRs* and *LILRB1*, which encode high-affinity receptors for HLA-C and HLA-G, respectively. The molecular signatures of dNK1 cells suggest their potential role in supporting fetal development. In contrast, dNK3 cells characterized by the expressions of *CD103, CD160*, and *KLRB1* display cytotoxic and immune-active signatures. Interestingly, both our study and others have reported a remarkable decrease in the abundance of the dNK1 subset while there is an increased proportion of the dNK3 subset in decidual tissues from RPL patients [16,17], suggesting that abnormal development of dNK subsets may contribute to early pregnancy loss. However, there is currently a lack of functional *in vivo* studies particularly investigating the pathophysiological roles played by these human dNK subsets in healthy and disordered pregnancies.

The properties of dNK cells significantly differ between humans and rodents, which hinders our understanding of the *in vivo* function of human dNK cells when using rodents as animal models. For instance, the MHC antigens on trophoblasts and MHC receptors on dNK cells are not homologous between humans and rodents, suggesting that human and rodent dNK cells may interact with their trophoblasts through distinct MHC-receptor mechanisms [18,19]. While mouse dNK cells (referred to as uterine NK or uNK in rodents) have limited effects on trophoblast cell differentiation, they can participate in uterine spiral artery remodeling [20]. The differences in characteristics between human and murine dNK cells make it challenging to directly correlate human dNK subsets with their murine counterparts, thereby limiting our understanding of the roles played by human dNK subsets in maintaining pregnancy. Therefore, it is necessary to develop a novel animal model that allows for *in vivo* studies on human dNK cells.

Humanized mouse models, in which human cells are transplanted into immunodeficient mice, have been proven to be valuable for investigating human cell development and roles in physiological and pathological processes [21–23]. The immuno-deficient NOG mouse strain (NOD/Shi-*scid*/IL-2Rγ^null^) lacks T and B cells completely, has minimal NK cell presence, and exhibits attenuated macrophage and dendritic cell activities [24]. These mice have been extensively utilized to study various aspects of human immune cell functions including CD34^+^ cells differentiation [24], chimeric antigen receptor-modified T cell (CAR-T) therapy for cancer [25], anti-tumor roles of NKT cells [26], as well as the development of human pNK cells [27]. However, there is little evidence regarding the suitability of NOG mice for transplantation studies involving human dNK cells and their functionality *in vivo*.

Based on the aforementioned evidence, we proposed to explore the pathophysiological functions of human dNK subsets from normal pregnant women or RPL patients at early gestational stage by adoptively transferring them into pregnant NOG mice. The pregnancy outcomes and fetal/placental development in recipient mice are closely associated with the characteristics exhibited by the donor's dNK subsets. Notably, our findings highlight that the CD56^bright^CD39^+^ dNK subset plays crucial roles in ensuring fetal survival through mechanisms that involve production of factors such as M-CSF, which modulates early differentiation processes of trophoblast stem cells. These insights shed light on the working mechanisms underlying human dNK cell subsets during pregnancy maintenance while also eliciting previously unappreciated targets for the therapeutic intervention against early pregnancy loss.

## RESULTS

### Phenotypic and functional distinctions of human dNK cells between normal pregnant women and RPL patients

**Figure 1.**
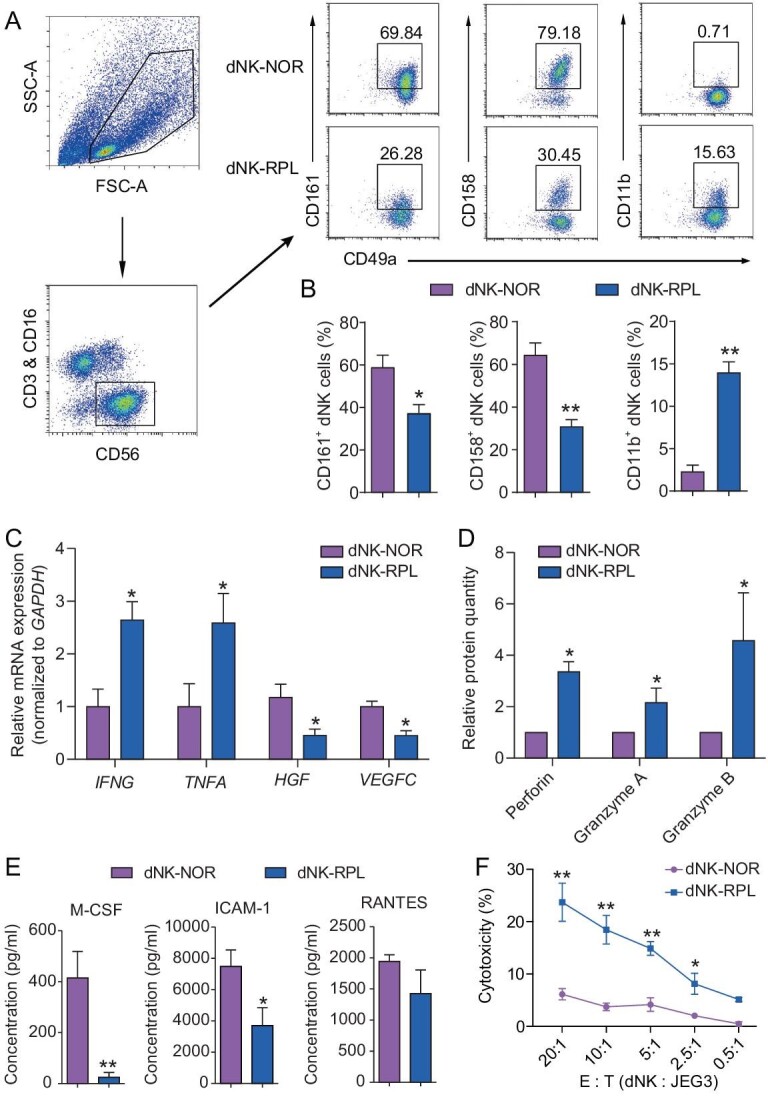
The cell properties of human dNK cells derived from normal pregnant women (dNK-NOR) and RPL patients (dNK-RPL) during gestational weeks 6–9. (A and B) Representative flow cytometry plots (A) and corresponding statistical analyses (B) demonstrating the frequency of functional receptors, including CD161, CD158, and CD11b in dNK-NOR (*n* = 6) and dNK-RPL (*n* = 6). (C) Quantitative real-time PCR analyses showing expression levels of pro-inflammatory cytokines (*IFNG* and *TNFA*) and growth factors (*HGF* and *VEGFC*) in freshly isolated dNK-NOR (*n* = 4) and dNK-RPL (*n* = 4). (D and E) Mass spectrometry analyses (D) and protein array analyses (E) showing levels of granule exocytosis proteins and cytokines/growth factors in the supernatants of dNK-NOR (*n* = 4) and dNK-RPL (*n* = 4) after *in vitro* culture for 48 hours. (F) Cytotoxicity assay showing the cytotoxic capacity of dNK-NOR and dNK-RPL against JEG3 cells. E: T, effector cell: target cell. The results are based on three independent experiments. All data are presented as mean ± SEM, with statistical analyses conducted using unpaired Student's *t*-test for panels B and E, multiple *t*-test for panels C and D, and two-way ANOVA with Fisher's LSD *post-hoc* test for panel F. Compared to dNK-NOR, *, *P* < 0.05; **, *P* < 0.01.

To compare the properties of dNK cells between normal and RPL pregnancies, we isolated CD3^−^CD16^−^CD56^+^ dNK cells from the decidual tissues of women with normal pregnancies or unexplained RPL at gestational week 6–9, referred to as dNK-NOR and dNK-RPL, respectively (Table 1), using magnetic activated cell sorting (MACS). Flow cytometry assay demonstrated >95% purity and >95% viability in the freshly isolated dNK cells (Fig. S1A in Supplementary data). The dNK cells possessed limited proliferative capacity but keep high viability for at least 48 hours when cultured *in vitro* (Fig. S1B). Upon frozen-thawed process, the cells could keep ∼80% viability (Fig. S1C). Flow cytometric analyses revealed significantly lower frequencies of immune inhibitory receptor CD161 and CD158 (the KIR recognizing HLA ligands) in dNK-RPL compared to dNK-NOR, along with a higher frequency of cytotoxicity-associated receptor CD11b (Fig. 1A and B). Real-time qPCR data showed elevated expression levels of inflammatory cytokines *IFNG* and *TNFA* in dNK-RPL while displaying decreased levels of growth factors *HGF* and *VEGFC* compared to dNK-NOR (Fig. 1C). To assess the secretory activities of dNK cells, purified dNK-NOR and dNK-RPL were cultured *in vitro* for 48 hours followed by collection of supernatants for mass spectrometry or protein array analyses. Mass spectrometry data demonstrated an over 2-fold increase in granule exocytosis proteins (Granzyme A, Granzyme B and Perforin) in dNK-RPL than that observed in dNK-NOR (Fig. 1D). Protein array assay results indicated significantly reduced levels of macrophage colony-stimulating factor (M-CSF/CSF-1), intercellular adhesion molecule-1 (ICAM-1), and RANTES (CCL5) in the supernatants of dNK-RPL as compared to that of dNK-NOR (Fig. 1E). Intriguingly, these three cytokines along with HGF and VEGFC have been reported to be regulators of trophoblast cell migration or invasion [13–15,17,28], suggesting potential defects within the ability of dNK-RPL to modulate trophoblast behaviors. Furthermore, the cytotoxicity assay showed that dNK-RPL exhibited significantly higher cytotoxicity against JEG3 (a human trophoblast cell line with HLA-G expression) and K562 leukemia cells compared to dNK-NOR (Fig. 1F and Fig. S2). These data collectively suggest that dNK cells in RPL patients retain unfavorable characteristics in regard to pregnancy maintenance.

**Table 1.** Clinical characteristics of the pregnant women enrolled in this study.

Category	Normal (*n* = 30)	RPL (*n* = 25)	*P* value
Ave age (range; year)	30.4 (24–39)	31.6 (26–39)	0.313
Ave No. of gravidities (range)	2.9 (1–5)	3.7 (3–6)	0.020
Ave No. of parities (range)	1.0 (0–2)	0.0 (0–0)	<0.001
Ave No. of miscarriages (range)	0.0 (0–0)	3.7 (3–6)	<0.001
Ave gestation (range; week)	6.9 (6–8)	7.4 (6–9)	0.112
Ave BMI (range)	21.6 (16.6–24.6)	22.0 (16.7–29.8)	0.315
Ave systolic pressure (range; mmHg)	110.4 (93–126)	111.1 (92–138)	0.856
Ave diastolic pressure (range; mmHg)	66.2 (56–79)	70.2 (57–83)	0.114
Smoker (%)	9.5 (2/30)	10.5 (2/25)	1.000

Note: *P* value in Smoker category was obtained by Chi-square test, while *P* values in all the other categories were obtained by unpaired Student's *t*-test. Ave, average; Normal, normal pregnancy; RPL, recurrent early pregnancy loss; BMI, body mass index.

### Adoptively transferred human dNK cells selectively enrich in the uterine environment and impact the pregnancy outcomes in NOG recipients

**Figure 2.**
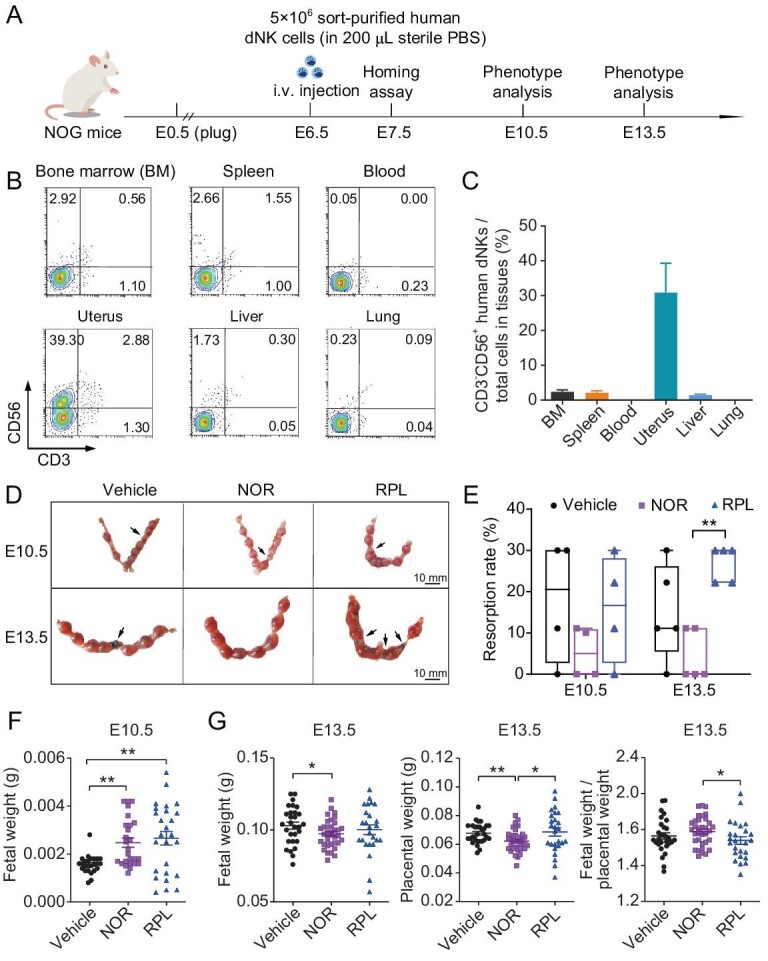
The pregnancy outcomes of NOG mice that received adoptive transfer of human dNK cells from normal pregnant women and RPL patients. (A) Schematic diagram illustrating experimental design of the adoptive transfer of 5 × 10^6^ human dNK cells isolated from normal pregnant women (NOR group) or RPL patients (RPL group), or an equal volume of sterile PBS (Vehicle group), into pregnant NOG mice at E6.5 via tail vein injection. (B and C) Representative flow cytometry plots (B) and corresponding statistical analysis results (C) demonstrating the distribution of transferred human dNK cells in multiple tissues of recipient mice at E7.5 (*n* = 2). (D) Representative images displaying implantation sites in recipient mice at E10.5 and E13.5, with aborted embryos indicated by black arrow. (E) Statistical analysis revealing abortion rates in recipient mice at E10.5 and E13.5. (F) Statistical results indicating fetal weight of surviving fetuses in recipient mice at E10.5. (G) Statistical analysis presenting fetal weight, placental weight, and ratio of fetal weight and placental weight of surviving fetuses in recipient mice at E13.5. Data presented in panels E–G are shown as mean ± SEM, and the statistical analyses were performed using unpaired Student's *t*-test. *, *P* < 0.05; **, *P* < 0.01.

To establish an *in vivo* mouse model supporting the functionality of human dNK cells, we intravenously transplanted isolated human dNK cells into pregnant immunodeficient NOG mice at embryonic day 6.5 (E6.5), a critical time point when uNK cell expansion occurs physiologically in the mouse uterus [29]. To reduce the influence of individual differences among dNK donors, dNK cells from 9–10 donors were pooled together (5 × 10^6^ cells per donor), and then 5 × 10^6^ pooled cells in 200 μL sterile PBS were injected into each mouse. After 24 hours, flow cytometry analysis revealed that the majority of transplanted human dNK cells were highly enriched in the mouse uterus, accounting for ∼30% of total uterine cells. However, fewer numbers of human dNK cells were detected in peripheral blood and other NK-gathering tissues of recipient mice including bone marrow, spleen, liver, and lung (Fig. 2A–C).

Upon observing the specific homing property of transplanted human dNK cells, we examined whether dNK cells can influence the progression of pregnancy in recipients. Pregnant NOG mice at E6.5 were randomly divided into three groups and intravenously injected with human dNK-NOR (NOR group, *N* = 9), human dNK-RPL (RPL group, *N* = 9), or equal volume of sterile PBS (Vehicle group, *N* = 9). At E10.5 and E13.5, which are key stages of fetal/placental development, phenotypic analysis was performed to assess fetal growth. Due to severe immune deficiency in NOG mice, the pregnancy rate and litter size were severely affected, as reflected by a median fetal resorption rate of 20.56% at E10.5 and 11.11% at E13.5 in the Vehicle group. Interestingly, transplantation of dNK-NOR significantly reduced fetal loss with a median fetal resorption rate of 0%–5% (5% at E10.5 and 0% E13.5, respectively). Strikingly, transplantation of dNK-RPL resulted in severe fetal lethality with a median fetal resorption rate reaching 30.0% at E13.5, which was 1.5-fold of that observed in the Vehicle group (Fig. 2D and E).

We quantified the weight of surviving embryos and their corresponding placentae in recipient mice. At E10.5, the surviving embryos in the Vehicle group exhibited relatively uniform sizes, while those in the NOR group were significantly larger with a mean fetal weight 1.5-fold of that in the Vehicle group (Fig. 2F). At E13.5, both the fetal and placental weight appeared slightly lower in the NOR group compared to the Vehicle group, possibly due to a larger litter size in NOR dams. Notably, significant heterogeneity was observed in embryo development within the RPL group, with some embryos displaying extremely low or high weights ranging from 0.0004 g to 0.0054 g at E10.5. This embryonic heterogeneity in the RPL group persisted until E13.5, and the average fetal/placental weight ratio was significantly lower than that of the NOR group (Fig. 2F and G), suggesting impaired nutrient transport efficiency in RPL placenta.

Taken together, these findings demonstrate that adoptive transplantation of human dNK cells from healthy or RPL pregnancies into NOG mice exerts profound effects on fetal and placental development in recipients, faithfully recapitulating pregnancy outcomes observed in their respective dNK donors’ pregnancies. These observations also suggest that this humanized mouse model is suitable for investigating functional aspects of human dNK cells within a physiological context.

### Transplantation of human dNK cells guides trophoblast differentiation towards both invasive and syncytial pathways in recipient mice

**Figure 3.**
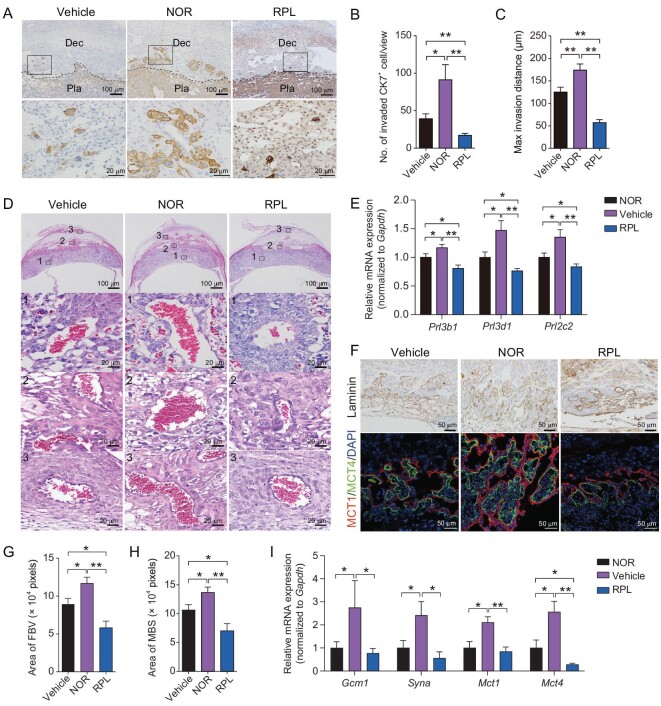
The placental phenotypes in NOG mice that received adoptive transfer of human dNK cells from normal pregnant women and RPL patients. (A) Representative images of immunohistochemical staining for cytokeratin 7 (CK7) in the placentae at E13.5 from the Vehicle, NOR, and RPL groups. Pla, placenta; Dec, decidua. The scale bar is set at 100 μm (top row) and 20 μm (bottom row). (B and C) Statistical analysis revealing the number of invaded trophoblasts (B) and their maximum invasion distance (C) in decidual tissues from each group. (D) Representative images of H&E staining of placentae at E13.5 from the Vehicle, NOR, and RPL groups. Higher magnification in bottom panels correspond to top panels highlighting specific areas such as canal vessel in labyrinth layer, proximal decidua, and distal decidua labeled as areas 1, 2, and 3, respectively. Scale bar is set at 500 μm (top row) and 20 μm (bottom three rows). (E) Quantitative real-time PCR analysis demonstrating marker gene expression related to trophoblast invasion in placentae at E13.5 from the Vehicle, NOR, and RPL groups. (F) Representative images depicting immunohistochemistry for laminin (top row) and immunofluorescence for MCT1 (red) and MCT4 (green) (bottom row) in the placentae at E10.5 from the Vehicle, NOR, and RPL groups, with DAPI (blue) indicating nuclei. Scale bar is set at 50 μm. (G and H) Statistical results representing the area of fetal blood vessels (FBV; G) and maternal blood sinuses (MBS; H) in the placentae at E10.5 from the Vehicle, NOR, and RPL groups. (I) Quantitative real-time PCR analysis showing expression of marker genes associated with trophoblast syncytialization in the placentae at E10.5 from the Vehicle, NOR, and RPL groups. Data are shown as mean ± SEM for panels B, C, E, and G–I. Statistical analyses were performed by one-way ANOVA with Fisher's LSD *post-hoc* test for panels B, C, G, and H; and multiple *t*-test for panels E and I based on number of the placentae *n* = 6 in the Vehicle group, *n* = 5 in the NOR group, *n* = 7 in the RPL group. For B, C, G, and H, one or two views from each placenta were analyzed. *, *P* < 0.05; **, *P* < 0.01.

The phenotype of fetal development in the recipient mice prompted us to further investigate the functional role of adoptive human dNK cells acting at the maternal-fetal interface. Considering the crucial roles of the placenta in supporting *in utero* growth of the fetus and the known ability of human dNK cells to promote trophoblast cell invasion [11–13], we initially assessed trophoblast invasiveness in NOG mice transplanted with human dNK cells. Immunohistochemistry for cytokeratin 7 (CK7) was performed at E10.5 and E13.5 to label trophoblasts at the maternal-fetal interface (Fig. 3A and Fig. S3A). As expected, there was a significant enhancement in trophoblast cell invasion in the NOR group compared to corresponding Vehicle controls, characterized by a notably increased number of invaded trophoblast cells within the decidua and enhanced invasion depth. Conversely, both invaded cell number and invasion depth of trophoblast cells were remarkably reduced in the RPL group compared to the NOR or Vehicle group (Fig. 3A–C and Fig. S3A–C). It has been established that uterine artery remodeling in pregnant mice is largely dependent on uNK cell accumulation along with their derived factors as well as the penetration by trophoblast giant cells (TGCs), which have analogous function to human EVT cells, and this intricate interplay results in expansion of the arterial lumen and subsequent augmentation of blood perfusion [30,31]. The results of H&E staining in the placentae at E13.5 revealed that arteries within proximal and distal decidua as well as canal in the placental labyrinth zone exhibited remarkably larger lumen size in the NOR group than the Vehicle controls; whereas arterial lumen diameter was considerably narrower in the RPL group when compared with the NOR or Vehicle group (Fig. 3D). Consistent with the phenotypical changes of trophoblast invasion, the qRT-PCR results revealed significantly higher expression levels of invasion-associated genes, including placental lactogen-2 (*Prl3b1/Pl-2*), placental lactogen-1 (*Prl3d1/Pl-1*), and proliferin (*Prl2c2/Plf*), in the placentae of the NOR group at E10.5 and E13.5 when compared with the Vehicle controls. However, these genes exhibited much lower levels in the RPL group than in either the NOR or Vehicle groups (Fig. 3E and Fig. S3D). Collectively, these findings indicate that transplanted human dNK cells actively participate in modulating trophoblast cell invasion and transformation of uterine arteries in recipient mice.

In both human and mouse placenta, the differentiation of syncytiotrophoblast (SynT) and subsequent branching of fetal blood vessels (FBVs) beneath the syncytial layers in the placental labyrinth zone are prerequisites for efficient nutrient transport from mother to fetus [32–34]. Considering the alterations in fetal weight/placental weight ratio observed in recipient NOG mice (Fig. 2G), we hypothesized that placental trophoblast syncytialization might also be influenced. General measurements of the area of placental labyrinth layer (Lab) and sponge layer (Sp) revealed obviously higher Lab/Sp ratios in NOR placentae relative to Vehicle controls, while this ratio was considerably lower in RPL placentae compared to the NOR or Vehicle group (Fig. S3E–F). Within the labyrinth layer, distinct identification of branched FBV and trophoblast-lined maternal blood sinusoid (MBS) was achieved through H&E staining and immunohistochemistry for laminin [35]. The two continuous layers of syncytiotrophoblast that primarily constitute the maternal-fetal exchange interface were separately labelled using Monocarboxylate Transporter 1 (MCT1; labeling SynT-I layer) and MCT4 (labeling SynT-II layer) [36]. Staining observations revealed that FBV branching and syncytial layer formation were markedly more extensive in the NOR group but markedly impaired in the RPL group compared to the Vehicle controls (Fig. 3F). Statistical analysis demonstrated that the FBV area was increased to ∼1.4-fold that of the Vehicle control level in NOR placentae whereas it was reduced to ∼65% of the Vehicle control level in RPL placentae (Fig. 3G). Additionally, MBS area increased by ∼30% in NOR group but decreased by ∼40% in RPL group compared to Vehicle controls (Fig. 3H), which was consistent with altered blood perfusion patterns within the placenta as shown in Fig. 3D. These data underscore the significant impact exerted by transplanted human dNK cells on placental transport efficiency.

The transcription factor glial cell missing-1 (GCM1, encoded by *Gcm1* gene) and fusogene Syncytin A (encoded by *Syna* gene) are well-established master regulators of mouse syncytiotrophoblast differentiation [37]. Consistent with the above histological findings, qRT-PCR results in the placentae revealed markedly increased expressions of *Gcm1, Syna, Mct1* and *Mct4* in the NOR group compared to the corresponding Vehicle controls. Conversely, a reduction in the expressions of these genes was found in the RPL group compared to either Vehicle or NOR groups (Fig. 3I). Collectively, these results indicate that adoptively transferred human dNK cells may play a significant role in regulating trophoblast syncytialization and consequently nutrient transport efficiency of placentae in NOG recipients.

### CD39^+^ dNK cells display features of supporting fetal growth and low cytotoxicity

**Figure 4.**
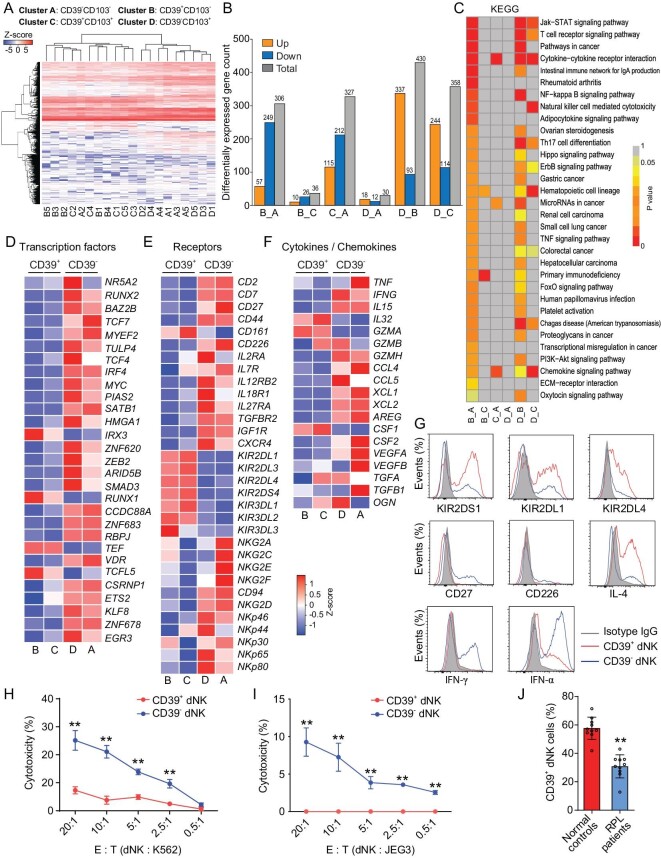
Transcriptomic assay and cell property analysis of human dNK subsets. (A) Heatmap with dendrogram analyses of Smart-seq2 RNA sequencing data showing the clustering characteristics of four flow-sorted dNK subsets from normal pregnant women at gestational week 6–9 (*n* = 5) using specific antibodies against CD3, CD16, CD56, CD39, and CD103. The CD3^−^CD16^−^CD56^+^ dNK cells were classified into four clusters based on the expression of CD39 and CD103: cluster A (CD39^−^CD103^−^), cluster B (CD39^+^CD103^−^), cluster C (CD39^+^CD103^+^), and cluster D (CD39^−^CD103^+^). (B) Two-by-two comparisons revealing differentially expressed genes among the four dNK clusters. (C) Two-by-two comparison-based KEGG enrichment analyses indicating signaling pathways associated with the differentially expressed genes among the four dNK clusters. (D–F) Heatmap displaying relative expressions (*Z*-score) of transcription factors (D), functional receptors (E), cytokines and chemokines (F) in the four dNK clusters. (G) Flow cytometric histograms demonstrating the expression of selected functional receptors and inflammation-associated cytokines in two subsets of dNK cells: CD16^−^CD56^+^CD39^+^ dNK subset (CD39^+^ dNK) and CD16^−^CD56^+^CD39^−^ dNK subset (CD39^−^ dNK). These subsets were isolated from five normal pregnant women. (H–I) Cytotoxicity assay showing the cytotoxic capacity of CD39^+^ dNK and CD39^−^ dNK cells against K562 (H) and JEG3 (I) target cells. The experiment was independently repeated three times using dNK cells from five normal pregnant women. (J) Flow cytometry results showing a difference in proportions of CD39^+^dNK cells between normal pregnant women (Normal; *n* = 10) and RPL patients (RPL patients; *n* = 10). Statistical analyses were performed by two-way ANOVA with Fisher's LSD *post-hoc* test for panels H–I and unpaired Student's *t*-test for panel J. *, *P* < 0.05; **, *P* < 0.01.

Through scRNA sequencing, both our and others’ studies have identified multiple subsets of human dNK cells [15–17]. CD39 and CD103 have been proposed as key molecules for characterizing the fetal-supporting dNK1 subset and immune-active dNK3 subset, respectively [15]. In our investigation of RPL patients, we observed a significant reduction in dNK1 subset but an increase in dNK3 subset in the decidua [17]. To further elucidate the properties of these dNK subsets, we sort-purified them from human normal decidua tissues (*n* = 5) at gestational week 6–9 using specific antibodies against CD56, CD16, CD3, CD39 and CD103 (Fig. S4). We successfully obtained four distinct clusters of CD3^−^CD16^−^CD56^+^ dNK: cluster A (CD39^−^CD103^−^), cluster B (CD39^+^CD103^−^, corresponding to the reported dNK1 subset), cluster C (CD39^+^CD103^+^), and cluster D (CD39^−^CD103^+^, corresponding to the reported dNK3 subset). Subsequently, smart-seq2 RNA sequencing was performed on these four cell clusters followed by bioinformatic analyses. Intriguingly, clusters B and C exhibited similar differential gene expression patterns as well as enriched KEGG pathways; whereas clusters A and D displayed identical transcriptomic features (Fig. 4A–C). These data suggest that clustering of dNK subsets based on predominant maker CD39 reflects their distinct functionalities. Indeed, heatmap analysis revealed substantial differences in transcription factors, receptors and cytokines/chemokines between two subpopulations: CD3^−^CD16^−^CD56^+^CD39^+^ dNK cells (referred to as CD39^+^; including clusters B and C), versus CD3^−^CD16^−^CD56^+^CD39^−^ dNK cells (referred to as CD39^−^; including clusters A and D) (Fig 4D–F). Flow cytometry analysis confirmed these transcriptomic findings by examining KIR and other activating/inhibitory receptors and several key cytokines in CD39^+^ and CD39^−^ dNK cells (Fig 4G). The CD39-dinstinguished dNK clusters also exhibited distinct cytotoxicity against K562 and JEG cells (Fig 4H and I), which was consistent with their gene expression profiles. Furthermore, the proportion of CD39^+^ dNK cells in RPL decidua was approximately half of that in normal pregnant controls (Fig 4J). Collectively, these findings suggest that human dNK cells can be functionally distinguished by the presence of CD39 and the CD3^−^CD16^−^CD56^+^CD39^+^ dNK cluster presents immunosuppressive and fetal-/placental-supporting characteristics.

### Human CD3^−^CD16^−^CD56^+^CD39^+^ dNK cells effectively rescue pregnancy failure and restore impaired placental development in dNK-RPL-transplanted NOG mice

**Figure 5.**
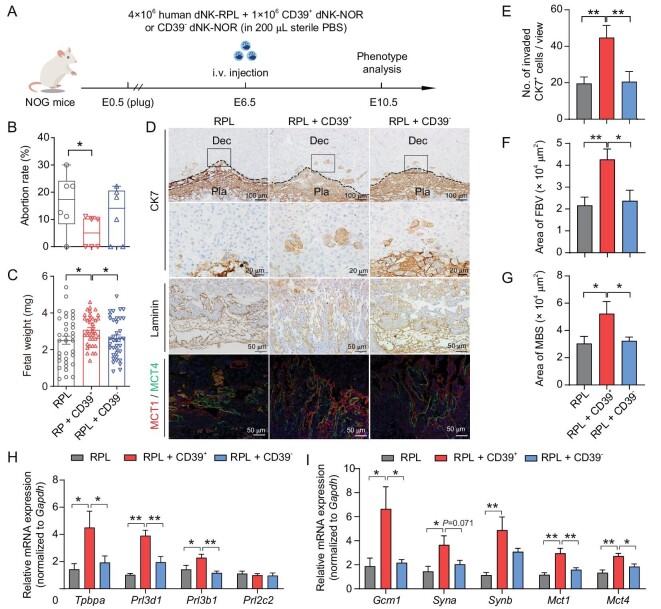
The pregnancy outcomes and placental phenotypes of NOG mice that received adoptive transfer of human dNK cells from RPL patients along with CD39^+^ dNK or CD39^+^ dNK from normal pregnancies. (A) Schematic diagram showing the experimental design of adoptive transfer of human dNK cells into NOG mice. 5 × 10^6^ dNK cells from RPL patients (RPL group), or 1 × 10^6^ CD39^+^ (RPL + CD39^+^) or CD39^−^ dNK cells (RPL + CD39^−^) from normal pregnancies along with 4 × 10^6^ dNK cells from RPL patients, were injected into pregnant NOG mice via the tail vein at E6.5. (B) Statistical analysis revealing abortion rates in recipient mice at E10.5, including the number of dams (*n*) as well as the ratio of aborted/total embryos (*r*). RPL group had *n* = 6 with *r* = 9/54; RPL + CD39^+^ group had *n* = 6 with *r* = 3/56; RPL + CD39^−^ group had *n* = 6 with *r* = 7/56. (C) Statistical analysis of fetal weight from surviving fetuses in recipient mice at E10.5. (D) Representative images of immunohistochemical staining for cytokeratin 7 (CK7, top two rows) and laminin (third row), as well as immunofluorescent staining for MCT1 (red) and MCT4 (green) (bottom row) in placentae at E10.5 from the RPL, RPL + CD39^+^, and RPL + CD39^−^ groups. DAPI (blue) was used to label nuclei. A black dash line indicated the boundary between decidua and spongiotrophoblast layer. Dec, decidua; Pla, placenta. Scale bar, 100 μm (top row), 20 μm (second row), and 50 μm (bottom two rows). (E) Statistical results showing the number of invaded trophoblast cells in decidual tissues from the RPL, RPL + CD39^+^, and RPL + CD39^−^ groups. (F and G) Statistical analysis revealing the area of fetal blood vessels (FBV; F) and maternal blood sinuses (MBS; G) in placentae at E10.5 from the RPL, RPL + CD39^+^, and RPL + CD39^−^ groups. (H and I) Quantitative real-time PCR analysis showing differential expression of marker genes associated with trophoblast invasion (H) and syncytialization (I) in placentae at E10.5 from the RPL, RPL + CD39^+^, and RPL + CD39^−^ groups. Data are shown as mean ± SEM for panels B, C, and E–I. Statistical analyses were performed by unpaired Student's *t*-test for panels B and C, one-way ANOVA with Fisher's LSD *post-hoc* test for panels E–G, and multiple *t*-test for panels H and I based on number of placentae *n* = 12 in the RPL group, *n* = 10 in the RPL + CD39^+^ group, *n* = 12 in the RPL + CD39^−^ group. For E, F, and G, one or two views from each placenta were analyzed. *, *P* < 0.05; **, *P* < 0.01.

To clarify whether the decreased proportion of CD39^+^ dNK cells in RPL decidua is involved in the occurrence of pregnancy failure, we performed adoptive transfer of 1 × 10^6^ CD39^+^ or CD39^−^ human dNK cells from normal pregnancy along with 4 × 10^6^ dNK cells from RPL patients (RPL + CD39^+^ group or RPL + CD39^−^ group), or 5 × 10^6^ dNK cells from RPL patients alone (RPL group), into NOG mice at E6.5, and compared their pregnancy outcomes and placental development at E10.5 (Fig. 5A). The transplantation of CD39^+^ dNK cells markedly reduced the high abortion rate in recipients of the RPL group, whereas CD39^−^ dNK cells had no favorable effect (Fig. 5B). The considerable heterogeneity in fetal weight observed in RPL dams was notably ameliorated by the transplantation of CD39^+^ dNK, resulting in a higher mean fetal weight compared to both the RPL and RPL + CD39^−^ groups (Fig. 5C). Immunostaining of CK7, laminin, MCT1 and MCT4 in the placental tissues revealed enhanced trophoblast cell invasion into the decidua, increased branching of fetal blood vessels and syncytial differentiation within the labyrinth zone, as well as extended areas of FBV and MBS in the RPL + CD39^+^ group relative to both the RPL and RPL + CD39^−^ groups (Fig. 5D–G). Consistently, qRT-PCR results demonstrated remarkably elevated expression levels of invasion-associated genes including *Prl3d1* and *Prl3b1*, as well as syncytialization-associated genes such as *Gcm1, Syna, Synb, Mct1* and *Mct4* in placentae from the RPL + CD39^+^ group compared to either RPL or RPL + CD39^−^ groups (Fig. 5H and I). However, the transplantation of CD39^−^ dNK cells showed little improvement on trophoblast differentiation (Fig. 5D–I). Collectively, these data provide evidence for the role of CD39^+^ dNK cells in supporting fetal/placental development and suggest that the loss of these cells may serve as a causative factor for RPL pathogenesis.

### Human CD3^−^CD16^−^CD56^+^CD39^+^ dNK cells regulate the early differentiation of trophoblast stem cells through production of cytokines including M-CSF

Subsequently, we aimed to explore how CD39^+^ dNK cells regulate trophoblast cell differentiation in recipient NOG mice. Considering the disparities in trophoblast-expressed MHC ligands between humans and rodents [31,38], it is unlikely that transplanted human dNK cells directly interact with recipient trophoblasts through MHC-receptor recognition; instead, they are more likely to exert their effects via paracrine signaling. To identify potential factors derived from human dNK cells that signal to trophoblasts, we integrated our scRNA-seq data with those from other studies on the human maternal-fetal interface during early gestation [15,17,39]. Amongst various genes encoding growth factors and cytokines, we found that M-CSF (encoded by *CSF1* gene) presents a strikingly specific expression in the dNK1 subset. The expression of its receptor, M-CSFR (encoded by *CSF1R* gene), was observed in some immune cell subsets as well as multiple trophoblast subsets including villous cytotrophoblasts (VCTs), syncytiotrophoblast (SCTs) and EVTs (Fig. S5A).

**Figure 6.**
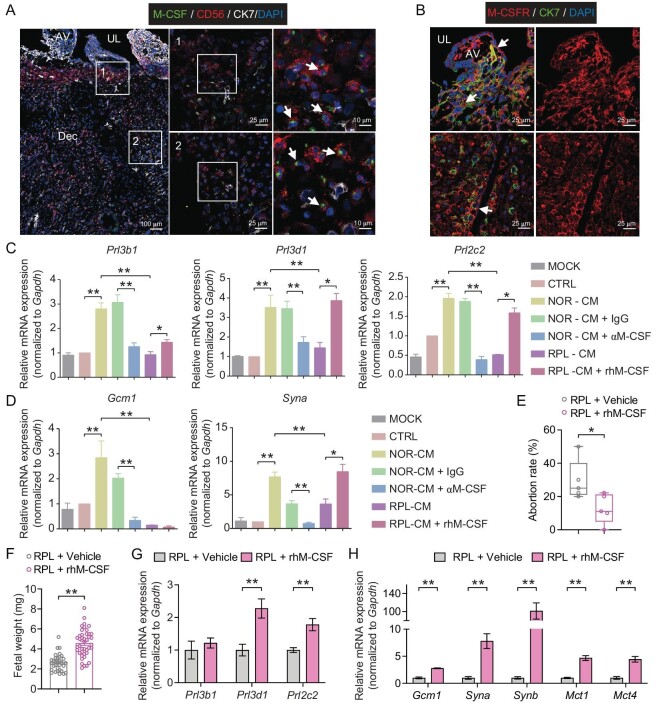
Effects of human dNK cells and M-CSF on mTSC differentiation and pregnancy outcomes. (A) Immunofluorescence staining for M-CSF (green), CD56 (red), and CK7 (white) at human maternal-fetal interface at gestational week 8. Middle panels represent higher magnification of the indicated region in left panels, and right panels represent higher magnification of the indicated region in middle panels. Regions 1 and 2 indicate decidual superficial layer and decidual compacta layer, respectively. The white arrows indicate CD56^+^M-CSF^+^ dNK cells. Scale bar, 100 μm (left panel), 25 μm (middle panels), and 25 μm (right panels). (B) Immunofluorescence staining for M-CSFR (red) and CK7 (green) at human maternal-fetal interface at gestational week 8. The white arrows indicate CK7^+^M-CSFR^+^ trophoblast cells. AV, anchoring villi; UL, uterine lumen; Dec, decidua. Scale bar, 25 μm. (C and D) Quantitative real-time PCR analysis showing expression of marker genes associated with trophoblast differentiation towards invasive pathway (C) or syncytial pathways (D) in mTSCs cells with various treatments as below. MOCK, mTSCs in differentiation media (inducing invasion in D–F, inducing syncytialization in G–H). CTRL, mTSCs in differentiation media supplemented with 50% dNK-free media. NOR-CM, mTSCs in differentiation media supplemented with 50% conditioned media of human dNK cells from normal pregnancies. NOR-CM-IgG, mTSCs in differentiation media supplemented with 50% conditioned media of human normal dNK cells that had been pre-incubated with pre-immune IgG. NOR-CM-αM-CSF, mTSCs in differentiation media supplemented with 50% conditioned media of human normal dNK cells that had been pre-incubated with neutralizing antibody against M-CSF. RPL-CM, mTSCs in differentiation media supplemented with 50% conditioned media of human dNK cells from RPL patients. RPL-CM-rhM-CSF, mTSCs in differentiation media supplemented with 50% conditioned media of RPL dNK cells and 1 ng/mL recombinant human M-CSF. (E and F) Pregnancy outcomes of NOG mice that were intravenously injected with 5 × 10^6^ dNK cells from RPL patients at E6.5 followed by intraperitoneal administration of 0.5 mg/kg/day of rhM-CSF (RPL + rhM-CSF, *n* = 5) or equal volume of sterile PBS (RPL + Vehicle, *n* = 5) for 4 days (E6.5–E9.5). Statistical analysis showing the abortion rates (E) and the fetal weight of surviving fetuses (F) in the recipient mice at E10.5. For panel F, RPL + Vehicle group, *n* = 32; RPL + rhM-CSF, *n* = 42. (G and H) Quantitative real-time PCR analysis showing the expression of marker genes associated with trophoblast invasion (G) and syncytialization (H) in the placentae at E10.5 from RPL + Vehicle (*n* = 10, 2 placentae/dam) and RPL + rhM-CSF (*n* = 10, 2 placentae/dam) groups. Data are presented as mean ± SEM. Data in panels C and D are from three independent experiments. Statistical analyses are performed by one-way ANOVA with Fisher's LSD *post-hoc* test for panels C and D, unpaired Student's *t*-test for panels E and F, and multiple *t*-test for panels G and H. *, *P* < 0.05; **, *P* < 0.01.

We further examined the distribution of M-CSF and M-CSFR at the maternal-fetal interface during early gestation in both humans and mice by immunohistostaining. Decidual tissues from normal human pregnancies at gestational week 7–9 were subjected to immunofluorescent staining for M-CSF or M-CSFR, as well as CK7 (to label trophoblasts) and/or CD56 (to label dNK cells). As depicted in Fig. 6A, M-CSF was mainly localized in CD56-positive dNK cells that were closely surrounded by trophoblasts both in the superficial layer (where trophoblast villi anchored) and the deeper compacta layer (where EVTs penetrated) of the implantation site (IS). Importantly, signals for M-CSFR were predominantly observed in VCT and SCT at the superficial layer as well as EVT that invaded into the deeper compacta layer (Fig. 6B). These findings are highly consistent with the scRNA-seq data presented in Fig. S5A. In mouse implantation sites from E6.5 to E10.5, specific signals of M-CSFR were also detected in distinct trophoblast subtypes including TGCs (on the invasive pathway of differentiation), chorionic plate trophoblasts and SynTs (on the syncytial pathway of differentiation) (Fig. S5B). Moreover, western blotting analyses showed that the protein level of M-CSF in RPL decidua was significantly lower than that in normal controls (Fig. S5C and D). This is consistent with the observation in Fig. 1E that dNK cells from RPL patients showed a remarkably decreased M-CSF production compared to normal dNK controls when cultured *in vitro*.

These results collectively indicate that human CD39^+^ dNK cells are a main source of M-CSF at the maternal-fetal interface and may play a considerable role in the regulation of trophoblastic differentiation towards invasive and syncytial pathways through interaction with M-CSFR. The impaired secretion of M-CSF by dNK cells in RPL patients may be associated with pregnancy failure in these individuals.

To elucidate the regulatory roles of M-CSF in trophoblast differentiation, we conducted *in vitro* experiments using mouse trophoblast stem cells (mTSCs) induced to differentiate towards either the invasive TGCs pathway or to the syncytial pathway, as described in Methods [40]. mTSCs were subjected to various treatments in two differentiation models, including dNK-free media (CTRL), conditioned media collected from human dNK cells of normal or RPL pregnancies after 24-hour culture (NOR-CM and RPL-CM), NOR-CM that had been pre-incubated with neutralizing antibody against human M-CSF (NOR-CM-αM-CSF) or with isotype-matched pre-immune IgG (NOR-CM-IgG), and RPL-CM that was supplemented with 1 ng/mL recombinant human M-CSF (RPL-CM-rhM-CSF). Following 48 hour treatments, qRT-PCR was performed to measure the genes indicating mTSC stemness and differentiation. The results showed a considerable reduction in the expression of stemness marker genes *Cdx2, Eomes* and *Esrrb* upon these treatments, indicating the initiation of mTSCs cell differentiation (Fig. S6A).

In the invasive differentiation model, the differentiated mTSCs exhibited significantly higher expression levels of invasion marker genes (*Prl3b1, Prl3d1, Prl2c2*) in the NOR-CM group compared to CTRL. Conversely, there were evidently decreased expressions in the RPL-CM group relative to the NOR-CM group. Interestingly, neutralizing M-CSF (NOR-CM-αM-CSF) completely blocked the enhanced expressions of *Prl3b1, Prl3d1* and *Prl2c2* in the NOR-CM group. On the contrary, adding rhM-CSF in RPL-CM (RPL-CM-rhM-CSF) efficiently restored the expressions of these genes to levels almost comparable to those in the NOR-CM group (Fig. 6C). The morphological observation of TGC differentiation revealed paralleled changes with gene expression patterns (Fig. S6B).

In the syncytial differentiation model, significantly promoted expressions of syncytial marker genes *Gcm1* and *Syna* were documented in the NOR-CM group while lower expressions of them were observed in the RPL-CM group compared to corresponding CTRL. The promotion of *Gcm1* and *Syna* expressions by NOR-CM was fully obstructed by abolishing M-CSF, while supplementing RPL-CM with rhM-CSF resulted in an evident rescue in *Syna* expression (Fig. 6D).

### Recombinant M-CSF efficiently reversed the exacerbated pregnancy failure in dNK-RPL-transplanted NOG mice

The above findings prompt us to further explore whether M-CSF itself is beneficial in restoring fetal growth in NOG mice transplanted with dNK-RPL. To test this, we administered recombinant human M-CSF (rhM-CSF) into the NOG mice that were adoptively transferred with dNK-RPL at E6.5 at a dose of 0.5 mg/kg/day from E6.5 to E9.5 (RPL + rhM-CSF group), with the administration of the same volume of sterile PBS as control (RPL + Vehicle group). Pregnancy outcomes and fetal development were assessed at E10.5. As expected, the administration of rhM-CSF effectively mitigated the increased abortion rate caused by transplantation of dNK-RPL (Fig. 6E). The fetal weight in RPL + hrM-CSF dams was significantly higher than that in RPL + Vehicle dams (Fig. 6F). Results of qRT-PCR revealed significant increase in both invasion- (Fig. 6G) and syncytialization-related (Fig. 6H) genes in the placentae of the RPL + hrM-CSF group compared to that of RPL + Vehicle group, suggesting that M-CSF can effectively promote placental trophoblast cell differentiation *in vivo*.

Taken together, these findings demonstrate that human dNK cells, particularly the CD39^+^ subset, play an essential role in guiding the early differentiation of trophoblast cells towards both invasive and syncytial pathways through producing cytokines such as M-CSF. The reduced proportion of CD39^+^ dNK subset and subsequent attenuated M-CSF production observed in RPL patients may account for impaired trophoblast differentiation, ultimately leading to fetal developmental retardation and pregnancy failure.

## DISCUSSION

Compared to rodents or even other primates, the invasion of human placental trophoblasts into the decidual stroma is significantly deeper and the trophoblast syncytialization is more extensive [41]. This phenomenon is believed to ensure sufficient blood supply and nutrient transportation to support the *in-utero* development of the large human brain [42]. Consequently, finding an appropriate animal model that can fully reflect the properties of the human maternal-fetal interface poses a significant challenge. Moreover, there are substantial differences in cell characteristics between human dNK cells and murine uNK cells [18,19], further complicating our understanding on the pathophysiological role of human dNK cells during gestation. The influence of human dNKs on placentation and associated pregnancy disorders has primarily been investigated through *in vitro* experiments and genetic studies focusing on allogeneic interactions between KIRs and MHC ligands [11–14]. In this study, we take advantage of highly immunodeficient NOG mice to establish a humanized mouse model, wherein adoptively transferred human dNK cells overwhelmingly homed to the uteri of pregnant NOG recipients. Furthermore, these adoptive human dNK cells make a significant impact on fetal/placental development in the recipients and faithfully recapitulate the pregnancy outcomes of the donors from whom the dNK cells were isolated. Notably, supplementation with CD3^−^CD16^−^CD56^+^CD39^+^ dNK cells or recombinant M-CSF, a cytokine that is predominantly produced by CD3^−^CD16^−^CD56^+^CD39^+^ dNK cells at the fetal-maternal interface, effectively mitigates fetal loss and placental defects in mice that received dNK cells from RPL patients. Overall, our study introduces a valuable tool for investigating the functions of human dNK cells and their subsets *in vivo* and demonstrates that CD56^+^CD39^+^ dNK cells play a pivotal role in promoting fetal and placental development at early stages of gestation through, at least in part, secreting M-CSF, which provides a novel insight on developing therapeutic strategies applicable to intervene in early pregnancy loss.

Here, the *in vivo* data from humanized NOG mice and the *in vitro* results from trophoblast stem cells collectively elucidate the pivotal role of human dNK cells in controlling trophoblast cell differentiation fate, encompassing both invasive and syncytial pathways. Proper trophoblast differentiation along the invasive pathway ensures adequate blood perfusion to the maternal-fetal interface, while appropriate trophoblast differentiation towards the syncytial pathway assists in the establishment of a suitable unit for nutrient access to the fetus. These two differentiation pathways synergistically contribute to optimal fetal development [32,43]. Numerous pieces of evidence support dNK cell-mediated enhancement of EVT differentiation and uterine vascular remodeling, primarily through the production of various cytokines and chemokines such as GM-CSF, M-CSF, HGF, VEGF, IL-8, IP-10, etc [11–14]. In this study, we unexpectedly observe a significant regulatory effect of human dNK cells on trophoblast differentiation towards the syncytial pathway, which is supported by evident changes in syncytial layer morphology and fetal blood vessel area within the placental labyrinth of NOG mice that received human dNK cells. This is further substantiated by transcriptional alterations in key genes associated with syncytialization. Comparable observations in an *in vitro* model of mTSC differentiation along the syncytial pathway after exposure to dNK condition media further validate that dNK cells play a regulatory role in trophoblast syncytialization. Undoubtedly, these effects are likely mediated via a paracrine signaling mechanism since villous trophoblasts are spatially separated from decidual NK cells [15]. Moreover, villous trophoblasts do not express MHC ligands that can be recognized by KIR receptors on dNK cells [44,45]. Our immunofluorescent assay at the human maternal-fetal interface demonstrates that a cluster of CD56^+^ dNK cells are localized in the proximal region of the implantation site [17], suggesting potential paracrine interactions between dNKs and villous trophoblasts. Indeed, we provide evidence that dNK-derived M-CSF efficiently promotes syncytial differentiation of mTSCs. Previous studies have suggested that various cell types including CD3^+^ T cells [46,47], dNK cells [46,48,49], endothelial cells and placental trophoblasts [50] as sources of M-CSF at the maternal-fetal interface. By analyzing multiple transcriptomic data and performing specific immune-staining at the human fetal-maternal interface, we confirm that dNK cells are the predominant source of M-CSF. In line with previous reports [51–53], we also observe widespread expression of M-CSF receptor on different subtypes of trophoblast at both human and murine maternal-fetal interface, indicating multifaceted functions for M-CSF in placental development. A previous study on primary human trophoblasts demonstrates that M-CSF stimulates the secretion of human chorionic gonadotropin (hCG) and human placental lactogen (hPL), two hormones exclusively produced by syncytiotrophoblasts [54]. Mice with a mutation in maternal *CSF1* gene (op/op) exhibit reduced implantation rate and fetal survival rate, which can be restored by administration of exogenous M-CSF [55]. In parallel, there have also been studies highlighting the promoting effect of M-CSF/M-CSFR signaling on EVT proliferation and differentiation [56]. It is noteworthy that myeloid cells such as macrophages, monocytes, and DC cells also express M-CSFR and there have been a wealth of studies demonstrating the pivotal role of M-CSF in regulating the pathophysiological function of myeloid cells through binding M-CSFR [57–59]. However, the NOG mice we utilized in this study are lacking myeloid cells [21,24], therefore the effects of either transplanted CD39^+^ dNK cells or the administrated exogenous M-CSF should be predominantly attributed to their regulatory roles on trophoblasts, but rarely in association with their interaction with myeloid cells at the maternal-fetal interface. Together, these findings suggest that dNK-produced M-CSF may act as a crucial factor to regulate diverse pathways involved in trophoblast differentiation to maintain placental health and fetal well-being. Affirmatively, in addition to M-CSF, it is worth exploring other paracrine factors derived from dNK cells that may coordinately control trophoblast syncytial differentiation.

Here we clarify that the CD56^+^CD39^+^ subtype is the predominant functional subset of human dNK cells responsible for regulating the fate of trophoblast cell differentiation. Our understanding of human dNK subtyping has primarily relied on transcriptome patterns revealed by scRNA-seq data [15–17]. In this study, both Smart-Seq2 RNA-seq analysis and adoptive transfer experiments involving human dNK subsets strongly support the pivotal role of CD39 in distinguishing functional human dNK cells. The gene expression signatures of CD39^+^ human dNK cells resemble those previously reported as dNK1 subset [15], and our *in vivo* findings in NOG mice confirmed their indispensable role in facilitating trophoblast differentiation towards distinct pathways, which is, at least in part, dependent on secretion of M-CSF. Notably, this and our previous study [17] consistently reveal a significant reduction (∼50%) in the proportion of CD39^+^ dNK cells in RPL patients as compared to normal pregnant women. Furthermore, supplementation with CD39^+^ dNK cells sufficiently rescues fetal loss and placental disorders observed in NOG mice that underwent adoptive transfer of RPL patient-derived dNK cells. These findings highlight a significant link between disturbed CD39^+^ dNK subset and adverse pregnancy outcomes. First, property analyses of dNK-RPL cells and CD39^+^ dNK cells indicate that the dysregulated cytokine production in dNK cells of RPL patients, for instance reduced M-CSF, can be largely attributed to the diminished number of CD39^+^ dNK cells. This cytokine disturbance not only impairs trophoblast cell differentiation but also disrupts interactions among various immune cell populations at the maternal-fetal interface [60]. Second, CD39^+^ dNK cells exhibit specific expression of diverse KIRs that can recognize HLA-C on EVTs. Although our NOG mouse model is not suitable for investigating direct dNK-EVT interactions due to the distinct MHC antigens between humans and rodents, which is a certain limitation of the present study, multiple *in vitro* studies have demonstrated the significant impact of KIR-HLA-C ligation on modulating active cytokine production by dNK cells [11,12,61]. Therefore, it is plausible to propose that a reduction in CD39^+^ dNK cells may be one of the key pathological factors contributing to maternal immunological rejection of the semi-allogenic fetus in RPL patients.

Apart from KIR-based direct interaction and cytokine-mediated indirect interaction with trophoblasts, CD39^+^ dNK cells may exert their functions through alternative mechanisms. The ATP-adenosine (ADO) axis is well known for its crucial role in regulating both innate and adaptive immunity [62,63]. Generally, extracellular ATP (eATP) is released from stressed or dying cells [64,65]. A high concentration of eATP can induce macrophage death, enhance the maturation and antigen-presenting capacity of dendritic cells (DCs) and augment the cytotoxic function of T and NK cells, thereby raising a pro-inflammation milieu [66]. As the rate-limiting ectoenzyme responsible for eATP degradation, CD39 collaborates with CD73 to convert extracellular ATP and ADP into AMP while generating adenosine to foster a local immunosuppressive environment [67,68]. Previous study has demonstrated that peripheral CD16^−^CD56^bright^ NK cells act as ‘regulatory cells’ by expressing high levels of ectoenzymes including CD39, CD73 and CD203a to produce ADO which inhibits autologous CD4^+^ T cell proliferation [69]. However, limited evidence exists regarding whether human dNK cells specifically expressing CD39^+^ regulate the maternal immune tolerance to the fetus through ATP-adenosine signaling pathways. The transcriptomic profile reveals that CD39^+^ dNK cells uniquely express some transcriptional factors such as IRX3, RUNX1, TCFL5, etc., warranting further investigation into how these transcription factors modulate the activation of ATP-adenosine signaling, as well as the immature status characterized by the absence of surface markers including CD11b and CD27.

In addition, our analyses on dNK properties comprehensively appreciate the feature of dNK cells in early pregnancy loss. For instance, we and others have reported increased dNK3 while lowered dNK1 proportion in RPL patients [16,17], whereas here we observed reduced frequency of CD161^+^ dNK cells (typically enriched in the dNK3 subset) in the patients. Our knowledge on the function of CD161 in dNKs remains largely lacking despite increasing reports on its potential roles in peripheral NK (pNK) cells. It has been demonstrated that CD161 is expressed by NK cells that are at early developmental stage where it may facilitate the cross-talk between NK cell precursors and other cell types within the bone marrow [70,71]. Within the periphery, down-regulation of CD161 may enhance the ability of NK cells to kill infected or transformed cells but would also result in unchecked cytotoxicity that could be harmful to ‘self’ [72,73]. Interestingly, CD161 expression in pNK cells is controlled by chromatin modifying transcription factor PLZF (encoded by *ZBTB16* gene) [74], and our scRNA-seq data on decidual leukocytes (GSA: HRA000237) revealed reduced expression of *ZBTB16* in dNK3 subset of RPL patients compared to that of normal pregnant controls (Fig. S7A). It is possible that the reduced expression of CD161 in dNKs of RPL patients may be due to the dysregulation of PLZF or other transcriptional factors, which leads to uncontrolled cytotoxicity and impaired response to cytokines (such as LLT1 from decidual stromal cells of extravillous trophoblasts) of dNK cells in the patients [15,75]. Another seeming paradox is that some dNK1-enriched factors including *PRF1, GZMA* and *GZMB* are higher in dNK-RPL, whereas the proportion of dNK1 subset is largely reduced in RPL patients [17]. Indeed, through reanalyzing our scRNA-seq data (GSA: HRA000237), we found higher expression levels of all these genes in various dNK subsets as well as the total dNK cells in RPL patients than in normal pregnant controls (Fig. S7B–D). All these findings highlight that the abnormalities of dNK cells in RPL patients are presented not only by the imbalance of dNK subsets in terms of proportion, but also by the dysfunction of various dNK subsets which may result from the dysregulation in transcriptional networks.

In conclusion, this study presents a valuable mouse model for investigating the paracrine function of human dNK cells at the maternal-fetal interface *in vivo*. The CD56^+^CD39^+^ dNK cell subset plays a predominant role in promoting trophoblast cell differentiation towards both invasive and syncytial pathways through, at least in part, secreting M-CSF. Our findings underscore the pivotal role of human dNK cells, particularly the CD39^+^ subpopulation, in controlling placental development and fetal health, providing novel insights on understanding the immune pathogenesis and developing novel therapeutic strategies for the treatment of recurrent early pregnancy loss.

## MATERIALS AND METHODS

For detailed materials and methods, please see the Supplementary data. The study protocol for human specimen collection was approved by the Ethics Committee at the Institute of Zoology, Chinese Academy of Sciences. Written informed consents were obtained from all subjects. All animal experiment procedures were approved by the Committee of Laboratory Animal Care and Ethics at Beijing Vital River Laboratory Animal Technology Co., Ltd and the Committee of Laboratory Animal Care and Ethics at the Institute of Zoology, Chinese Academy of Sciences.

## Supplementary Material

nwae142_Supplemental_File
