# Supplementary material for: Human CD56+CD39+ dNK cells support fetal survival through controlling trophoblastic cell fate: immune mechanisms of recurrent early pregnancy loss
Source: Natl Sci Rev. 2024 Apr 11;11(6):nwae142. doi: 10.1093/nsr/nwae142 (PMC11223582; doi:10.1093/nsr/nwae142)
Supplement: nwae142_Supplemental_File [file nwae142_supplemental_file.docx]

**Supplementary Data for**

Human CD56^+^CD39^+^ dNK cells support fetal survival through controlling trophoblastic cell fate: immune mechanisms of recurrent early pregnancy loss

Running title: CD56^+^CD39^+^ dNKs support fetal/placental growth

Wentong Jia^1,#^, Liyang Ma^1,#^, Xin Yu^1,7,#^, Feiyang Wang^1,2,7^, Qian Yang^4^, Xiaoye Wang^2^, Mengjie Fan^2^, Yan Gu^5^, Ran Meng^6^, Jian Wang^4^, Yuxia Li^1^, Rong Li^2,*^, Xuan Shao^1,3,7,*^, and Yan-Ling Wang^1,3,7,*^

^1^State Key Laboratory of Stem cell and Reproductive Biology, Key Laboratory of Organ Regeneration and Reconstruction, Institute of Zoology, Chinese Academy of Sciences, Beijing 100101, China; ^2^National Clinical Center for Obstetrics and Gynecology, Peking University Third Hospital, Beijing 100191, China; ^3^Beijing Institute for Stem Cell and Regenerative Medicine, Beijing 100101, China; ^4^NHC Key Lab of Reproduction Regulation, Shanghai Engineering Research Center of Reproductive Health Drug and Devices, Shanghai Institute for Biomedical and Pharmaceutical Technologies, Shanghai 200237, China; ^5^Department of Family Planning, The Second Hospital of Tianjin Medical University, Tianjin 300211, China; ^6^Department of Prenatal Screening, Haidian Maternal and Child Health Hospital, Beijing 100080, China; ^7^University of the Chinese Academy of Sciences, Beijing 101408, China.

# These authors contribute equally to this study.

* Correspondence: Yan-Ling Wang ([wangyl@ioz.ac.cn](mailto:wangyl@ioz.ac.cn)), Xuan Shao ([shaoxuan@ioz.ac.cn](mailto:shaoxuan@ioz.ac.cn)) or Rong Li ([roseli001@sina.com](mailto:roseli001@sina.com)).

**This PDF file includes:**

Materials and Methods

Supplementary Figures 1 to 7

Supplementary Tables 1 to 2

**MATERIALS AND METHODS**

**Human sample collection and Ethic permission**

The study protocol for human specimen collection was approved by the Ethics Committee at the Institute of Zoology, Chinese Academy of Sciences. Written informed consents were obtained from all subjects.

Clinical samples of villous and decidual tissues from normal (n=30) or unexplained RPL (n=25) pregnancies at gestational week 6−9 were collected following therapeutic termination of pregnancy in Peking University Third Hospital, the Second Hospital of Tianjin Medical University, or Beijing Haidian Maternal and Child Health Hospital. All samples were subjected to cell isolation or tissue fixation within 1 hour after surgery.

Unexplained early stage RPL was diagnosed when subjects suffered three or more consecutive spontaneous loss of clinical intrauterine pregnancy before 10 weeks of gestational age, which was conﬁrmed by ultrasound scan^1-3^. Women with any endocrine disorders, fetal chromosomal or congenital abnormalities, uterine anatomical disorders, renal disease, or pregnancies conceived any fertility treatments were excluded from this study. In addition, all enrolled RPL patients received comprehensive medical care during pregnancy and underwent pregnant termination within one week after cessation of fetal heartbeat. The clinical characteristics of participants are summarized in Table 1.

**Animals**

NOG (NOD/Shi-*scid*/IL-2Rγ^null^) mice were purchased from Beijing Vital River Laboratory Animal Technology Co., Ltd and housed in the germ-free animal care facility with a fixed 12h/12h light/dark cycle. All mice had *ad libitum* access to food and water. Virgin female mice (8-12 weeks old) were mated with fertile males (10 weeks old) and the morning of the date when the vaginal plug was detected was recorded as embryonic day (E) 0.5. Mice were euthanized for tissue harvest by using carbon dioxide asphyxiation. All procedures were approved by the Committee of Laboratory Animal Care and Ethics at Beijing Vital River Laboratory Animal Technology Co., Ltd and the Committee of Laboratory Animal Care and Ethics at Institute of Zoology, Chinese Academy of Sciences.

**Isolation of human decidual NK cells**

The isolation of human decidual NK cells was performed according to previously described methods^4^. In brief, fresh human decidual tissues from patients at gestational week 6-9 were rinsed with cold sterile PBS and then homogenized with gentleMACS Dissociator (Miltenyi Biotec). The tissue was digested in RPMI-1640 media (Gibco) containing 1 mg/mL type IV collagenase (Gibco) and 10 U/mL DNase I (Sigma-Aldrich) at 37°C for two 30-minute rounds. The cell suspensions were sequentially filtered through 100-, 70-, and 40-μm cell strainers (BD-Falcon), followed by centrifugation at 1,000 rpm for 10 min. The cell pellets were washed twice with RPMI-1640 media and re-suspended in RPMI-1640 media supplemented with 10% fetal bovine serum (FBS; Gibco), as well as Penicillin-streptomycin solution at concentration of 100 U/mL each. Subsequently, the cells were plated into 150-mm petri dishes and incubated in a humidified incubator with 5% CO_2_ at 37°C for 2 hours. Non-adherent cells were harvested and subjected to Ficoll-Paque Plus (GE Healthcare)-based mononuclear cell separation. After washing with PBS, the mononuclear cells were subjected to magnetic activating cell sorting (MACS) to purify CD3^-^CD16^-^CD56^+^ dNK cells using NK Cell Isolation Kit (Cat#: 130-092-657, Miltenyi Biotec). Flow cytometric analyses were conducted to verify the cell purity. The typical yield of dNK cells was approximately 6 x 10^5^ cells per gram of decidual tissues. To isolate specific subsets of human dNK cells, the separated mononuclear cells stained with fluophor-conjugated specific antibodies against CD3, CD16, CD56, CD39 and CD103 were subjected to fluorescence-activated cell sorting (FACS), with detailed methods for staining in the Flow cytometry and cell sorting section. For collection of conditioned media, purified dNK cells were culture at a density of 2 × 10^6^/mL in dNK culture media (RPMI-1640 media supplemented with 10% FBS, 100 U/mL Penicillin and Streptomycin each, and 5 ng/mL recombinant human IL15 (Cat#: 200-15-2, Peprotech)). The supernatants were collected following 48h of culture, centrifuged at 16,000 × g for 10 min to remove cell debris and frozen at -80°C.

**Flow cytometry and cell sorting**

For flow cytometry, cells were washed with 1 mL PBS and centrifugated at 400 x g for 5 min at 4°C. Cells were then stained with Helix NP^TM^ NIR (1:1000, Cat#: 425301, Biolegend) at 4°C for 5 min followed by two times of wash with PBS. The cell pellets were re-suspended in 100 μL FACS buffer (PBS supplemented with 5% FBS) containing 1% PerCP-Cy5.5-conjugated mouse anti-human CD16 (Cat#: 302027, Biolegend) and incubated at 4°C for 20 min. After two times of wash with FACS buffer, the cells were stained with the primary antibodies diluted (1:200) in 100 μL FACS buffer and incubated at 4°C for 30 min while protected from light. The cells were then washed twice with FACS buffer and re-suspended in 300 uL FACS buffer for flow cytometry or cell sorting.

For intracellular cytokine staining, the dNK cells purified by MACS were cultured in dNK culture media containing 50 ng/mL PMA (Sigma-Aldrich), 250 ng/mL Calcium Ionophore (Sigma-Aldrich), and 10 μg/mL monensin sodium salt (Sigma-Aldrich) for 4 hours at 37°C. The cells were then washed twice with PBS and then subjected to surface staining of CD3, CD56, CD39, and CD103. After wash with FACS buffer, the cell pellets were re-suspended in 250 μL Cytofix/Cytoperm solution (BD Biosciences) and kept in dark for 20 min at 4°C. Cells were wash twice with 1 mL Perm/Wash buffer (BD Biosciences) and re-suspended in 100 μL Perm/Wash buffer containing intracellular cytokine antibodies. After a 30-minute incubation at 4°C, cells were washed twice with 1 mL Perm/Wash buffer and finally re-suspended in 300 μL Perm/Wash buffer for flow cytometry. CytoFLEX Flow Cytometer (Beckman) were utilized for flow cytometry and MoFlo XDP Cell Sorter (Beckman) were applied for cell sorting. The flow cytometric analyses were carried out with CytExpert (Beckman) or FlowJo v10. Primary antibodies used for flow cytometry are listed in Supplemental Table 2.

**Adoptive transfer of human dNK cells into NOG mice**

The pregnant NOG mice at E6.5 were subjected to adoptive transfer of human dNK cells. To minimize inter-individual differences among human donors, dNK cells from 9-10 donors (5×10^6^ cells per donor) were pooled together and intravenously injected into individual pregnant NOG mouse with 5×10^6^ pooled cells in 200 μL sterile PBS per mouse. The mice were randomly assigned to the following groups: Vehicle (injected with 200 μL sterile PBS; n=9), NOR (transferred with human dNK cells from normal pregnancies; n=12), RPL (transferred with human dNK cells from RPL patients; n=9). Two mice from the NOR group were sacrificed at E7.5 for flow cytometric analyses of the distribution of adoptively transferred human dNK cells in peripheral blood, bone marrow, spleen, uterus, liver, and lung tissues based on the staining of PerCP-Cyanine5.5 anti-human CD3 (Cat#: 45-0037-42, eBioscience) and APC anti-human CD56 antibody (Cat#: 318310, Biolegend). At E10.5 and E13.5, four or five mice from each group were sacrificed to collect fetuses and placentae collection for further analyses.

In the experiments investigating different subsets of dNK cells, pregnant NOG mice at E6.5 were randomly divided into the following groups: RPL group (transferred with 5×10^6^ human dNK cells from RPL pregnancy; n=6), RPL+CD39^+^ group (transferred with 1×10^6^ CD39^+^ human dNK cells from normal pregnancy along with 4×10^6^ dNK cells from RPL patients; n=6), RPL+CD39^-^ group (transferred with 1×10^6^ CD39^-^ human dNK cells from normal pregnancy along with 4×10^6^ dNK cells from RPL patients; n=6). At E10.5, the mice were sacrificed to collect fetuses and placentae for further analysis.

In the experiments of recombinant human (rh)M-CSF administration, pregnant NOG mice were randomly divided into two groups: RPL + rhM-CSF group (transferred with 5×10^6^ human dNK cells from RPL patients at E6.5 followed by intraperitoneal administration of rhM-CSF at a dose of 0.5mg/kg/day from E6.5 to E9.5; n=5); RPL+Vehicle group (transferred with 5×10^6^ human dNK cells from RPL patients at E6.5, followed by intraperitoneal administration of equal volume of sterile PBS from E6.5 to E9.5; n=5). Mice were sacrificed at E10.5 and fetuses and placentae were harvested for further analyses.

**Histological analyses**

Fresh tissue samples were fixed in 4% paraformaldehyde (PFA; Sigma-Aldrich) at 4°C for 10 hours. The fixed tissues were subjected to gradient ethanol dehydration, tissue clear in xylene and paraffin embedding, or gradient sucrose dehydration and embedding in OCT compound (Tissue Tek).

The paraffin-embedded tissues were sectioned at 5 μm using paraffin microtome (Leica). Following routine dewaxing, rehydration, endogenous peroxidase inactivation, and heat-induced antigen retrieval in citrate buffer, the sections were blocked with 3% BSA (Sigma-Aldrich) and incubation with primary antibodies against Cytokeratin 7 (CK7; Cat#: ab181598, Abcam), Laminin (Cat#: L9393, Sigma-Aldrich), or M-CSFR (Cat#: ab183316, Abcam) overnight at 4°C. After wash with PBS, the sections were incubated with HRP-conjugated secondary antibody (Cat#: PV-9001, ZSGB-Bio) for 1 hour at room temperature. DAB substrate (ZSGB-Bio) were then used to visualize the signals.

The OCT-embedded tissues were sectioned at 10 μm by cryo-microtome (Leica). The sections were mounted on Super Frost Plus slides (VWR), dried out on hot plate at 55°C for 5 min, fixed in 4% PFA for 5 min. Following three times of wash with PBS, the sections were subjected to heat-induced antigen retrieval in citrate buffer and incubated overnight with primary antibodies against MCT1 (Cat#: AB1286, Millipore), MCT4 (Cat#: AB3314P, Millipore), NCAM1/CD56 (Cat#: ab75813, Abcam), CK7 (Cat#: ab181598, Abcam), M-CSF (Cat#: AF216, R&D systems), or M-CSFR (Cat#: ab183316, Abcam) at 4°C. The secondary antibody incubation was performed by using corresponding IgG conjugated with Alexa Fluor 488, Alexa Fluor 594 or Cy5 dyes (Jackson ImmunoResearch Laboratories) at room temperature for 1 hour. After nuclei staining with 4,6-diamidino-2-phenylindole (DAPI; Sigma-Aldrich), the sections were mounted with mounting media. Images were captured under Zeiss LSM 780 laser confocal microscope and analyzed by ZEN Microscopy Software (Zeiss).

ImageJ was utilized to measure the areas of fetal blood vessels (FBV) and maternal blood sinusoid in placental labyrinth (Lab) layer based on the images from H&E staining and IHC staining for Laminin. Specifically, vessels that are stained positive for Laminin and contain nucleated red blood cells were defined as FBV while those contain non-nucleated red blood cells were considered as MBV. Then all the FBV and MBV in the view were circled and their areas were individually measured by ImageJ. In each indicated experimental group, 5-7 placentae with 1-2 views from each were analyzed.

**Cytotoxicity assay**

The cytotoxicity assay was performed using CytoTox 96® Non-Radioactive Cytotoxicity Assay Kit (Cat#: G1780, Promega), which is based on quantitatively measuring lactate dehydrogenase (LDH), a stable cytosolic enzyme that is released upon cell lysis. In brief, the freshly isolated dNK cells were co-cultured with target cells (K562 or JEG3) in 96-well plates at different ratios of effector cell: target cells (E:T) for 4 h at 37°C. The supernatants were incubated with substrate and the absorbance was recorded at 490 nm or 492 nm. The percent cytotoxicity for each E:T cell ratio was calculated according to the manufacturer's instruction.

**Cell proliferation assay**

Cell counting kit-8 (Cat#: HY-K0301, MedChem Express) was utilized to examine the proliferative capacity of human dNK cells. Briefly, 1 × 10^5^ freshly isolated dNK cells from normal pregnancies were plated into each well of a 96-well plate and incubated in a humidified incubator with 5% CO_2_ at 37°C. The wells with cell-free culture media serve as blank control for background subtraction. At 0 h, 24 h, and 48 h of culture, 10 μl of the CCK-8 solution was added into each well of the plate followed by a 2-hour incubation at 37°C. The absorbance at 450 nm was then measured using a microplate reader.

**Culture and differentiation of mTSCs**

The mTSCs originated from CD-1 mouse were generously provided by Dr. Haibin Wang at Xiamen University, China, and were cultured according to the established protocol^5^. The cells were maintained in TSC complete culture media consisting of 70% mouse embryonic fibroblasts-conditioned medium (MEF-CM), 30% TSC media (RPMI1640 supplemented with 20% fetal bovine serum, 2 mM L-glutamine, 1 mM sodium pyruvate, 100 U/mL Penicillin-Streptomycin, and 55 μM β-mercaptoethanol), along with the addition of 25 ng/mL FGF4 (PeproTech) and 1 μg/mL heparin (Sigma-Aldrich). To induce differentiation of mTSCs towards an invasive pathway, MEF-CM, FGF4 and heparin were withdrawn from the culture media. Instead, cell differentiation along the syncytial pathway was induced by supplementing with 3 μM CHIR 99021 (Sigma-Aldrich) ^6,7^. Under these differentiation condition aimed at investigating the impact of dNK cells on mTSCs behavior, various treatments were applied: exposure to either a mixture of M-CSF neutralizing antibody (Cat#: AF216, R&D Systems) or IgG control (Cat#: AB-108-C, R&D systems) in combination with dNK-NOR conditioned media at a concentration of 50%, or treatment with recombined human M-CSF (rhM-CSF, R&D systems) in combination with dNK-RPL conditioned media at a concentration of 50%. Cell morphology was documented during a period of incubation lasting for up to 48 hours before harvest for subsequent RNA extraction.

**Protein array**

The protein levels of cytokines in the culture media of human dNK cells were measured using Quantibody Human Inflammatory Factors Array-3 (QAH-INF-G3, Raybiotech) according to the manufacturer's instructions. This array allows to screen up to 40 human cytokines simultaneously.

**Mass spectrometry analysis**

The relative protein quantification in the supernatants of human dNK cells was performed using Mass spectrometry analysis according to a previously reported method^8^. Briefly, the protein concentration was adjusted to 1 mg/mL, and 100 μL media were subjected to reduction and alkylation with 4 mM DTT and 8 mM IAA, respectively. The beads were washed with TEAB and re-suspended in 100 mM TEAB for trypsin digestion. The reaction was carried out overnight at 37°C with agitation. After digestion, the samples were desalted, dried, and reconstituted in 100 μL of 100 mM TEAB. Dimethyl labeling was performed by adding wither 4 μL of 4% (vol/vol) CH_2_O or ^13^CD_2_O to label the peptides in the sample. Finally, the labelled samples were mixed and centrifuged, followed by addition of 4 μL of 0.6 M NaBH_3_CN for incubation at room temperature with agitation for 1 hour. The reaction was quenched by adding ammonia and formic acid prior to consecutive LC-MS/MS analysis. The different dimethyl isotope labels were considered as variable modifications on peptide N termini and lysine residues. The monoisotopic mass increment for light-labelled proteins from dNK-NOR supernatants and heavy-labelled proteins from dNK-RPL supernatants are 28.0313Da and 36.0757Da, respectively. Quantitation was performed using CIMAGE software to calculate the abundance ratio of heavy-labeled proteins to light-labeled proteins, representing the relative protein abundance in the supernatants of dNK-RPL to that of dNK-NOR.

**Western blotting**

Western blotting was performed following the previous protocols^4^. Briefly, total proteins from cultured cells or tissues were extracted and 40 μg protein was loaded into to a 10% SDS-PAGE gel for electrophoresis and then electro-transferred onto a PVDF membrane (Millipore). The membrane was blocked with 5% BSA for 1 hour at room temperature followed by overnight incubation with primary antibodies against human M-CSF (Cat#: AF216, R&D systems) or GAPDH (Cat#: AB1019P, AmeriBiopharma) at 4°C. After washing with TBST, membranes were incubated with horseradish peroxidase (HRP)-conjugated secondary antibodies (Jackson ImmunoResearch Laboratories) for 1 hour at room temperature. The signals were then visualized with Thermo Supersignal West Pico chemiluminescent substrate (Thermo Fisher Scientific) and analyzed by using Chemiluminescence imaging based GeneGnome XRQ (Syngene). The relative intensity of each specific molecule was calculated by normalizing the blot intensity of M-CSF to GAPDH from the corresponding blot.

**RNA extraction and Quantitative real-time PCR**

Total RNAs from cultured cells or frozen tissues were extracted and reverse transcribed into cDNA following the previously reported protocol^4^. The cDNA was subjected to quantitative real-time PCR (qRT-PCR) using a Lightcycler (Roche) in the presence of reaction mixture contained SYBR Green PCR mix (Takara) and 10 pM of primers. All primer sequences were listed in Supplementary Table 1. All PCR reactions were performed in triplicates, and the relative mRNA expression levels were determined using the 2^-ΔΔCT^ method^9^ with normalization to GAPDH.

**RNA Sequencing and Data analysis**

Four subsets of human dNK cells were flow sorted, including CD39^+^CD103^+^, CD39^+^CD103^-^, CD39^-^CD103^+^, and CD39^-^CD103^-^. Two hundred cells from each subset were directly sorted into PCR tubes containing lysis components and ribonuclease inhibitor. Subsequently, Smart-Seq2-based RNA sequencing was performed on Illumina Hiseq platform (Annoroad Gene Technology Co. Ltd.). Briefly, an Oligo-dT primer was incorporated in the reverse transcription reaction for first-strand cDNA synthesis followed by PCR amplification to enrich the cDNA. The resulting product underwent magbeads purification to remove impurities. The cDNA production was assessed using Qubit® 3.0 Fluorometer and Agilent 2100 Bioanalyzer to ensure that it had an expected length of around 1~2 kbp. Qualified cDNAs were randomly sheared using ultrasonic waves and subjected to a standardized Illumina library preparation protocol which included DNA fragmentation, end repair, 3’ ends A-tailing, adapter ligation, PCR amplification and library validation steps. Following cDNA library preparation, PerkinElmer LabChip®GX Touch and Step OnePlus™ Real-Time PCR System were employed for library quality inspection. Finally, qualified libraries underwent PE150 sequencing on the Illumina Hiseq platform.

The quality control of the sequenced raw data involved filtering out contaminated reads containing adapters (Read Bases contained more than 5bp of adapter sequences), low-quality reads (Reads Bases with a phred Quality value less than or equal to 19 accounting for more than 15%), and reads containing N base exceeding 5% of total bases. The filtered clean data was mapped to the reference genome using TopHat v2.0.12 and Bowtie2, while gene and genome annotation files were obtained from the UCSC Genome Browser (http://hgdownload.soe.ucsc.edu/goldenPath/galGal4) and used to build the reference genome library via Bowtie2 v2.2.3. Mapping results were visualized using Integrative Genomics Viewer (IGV) in heatmap, histogram, scatter plot or other graphs formats, while HTSeqv0.6.0 was employed to calculate gene counts and Reads Per Kilobase of exon model per Million mapped reads (RPKM) used for assessing expression levels.

The RPKMs were imported into the R/Bioconductor package DESeq for differentially expressed gene expression analysis. The significance of the differentially expressed genes was determined by pairwise comparisons between all biological replicates of the enriched/enhanced tissue (or group of tissues) and all other tissues (or groups). The multiple-testing adjusted P-value (FDR 5%) was used to assess the statistical significance of differential gene expression. Differentially expressed genes were further analyzed for Gene Ontology (GO) enrichment using Blast2go and Kyoto Encyclopedia of Genes and Genomes (KEGG) pathway enrichment using KAAS. Heatmaps depicting selective genes, including transcriptional factors, cytokines and chemokines, and receptors et al., were generated using the heatmap.2 in the R/Bioconductor gplots package.

**Statistical analyses**

All statistical analyses were performed using GraphPad Prism 8.0 (GraphPad Software). Data are presented as mean ± standard error of the mean (SEM). Differences among groups were analyzed using Unpaired Student’s *t*-test, multiple *t*-test, One-way ANOVA with Fisher’s LSD post-hoc test, or two-way ANOVA with Fisher’s LSD post-hoc test depending on the experimental group numbers, comparisons, and test requirements. Statistically significance was considered as *P*-value < 0.05.

**REFERENCES**

1 Rai, R. & Regan, L. Recurrent miscarriage. *The Lancet* **368**, 601-611 (2006). <https://doi.org:10.1016/s0140-6736(06)69204-0>

2 Quenby, S. *et al.* Miscarriage matters: the epidemiological, physical, psychological, and economic costs of early pregnancy loss. *Lancet* **397**, 1658-1667 (2021). <https://doi.org:10.1016/S0140-6736(21)00682-6>

3 Strobel, L. *et al.* Different Background: Natural Killer Cell Profiles in Secondary versus Primary Recurrent Pregnancy Loss. *J Clin Med* **10** (2021). <https://doi.org:10.3390/jcm10020194>

4 Ma, L. *et al.* dNK cells facilitate the interaction between trophoblastic and endothelial cells via VEGF-C and HGF. *Immunol. Cell Biol.* **95**, 695-704 (2017). <https://doi.org:10.1038/icb.2017.45>

5 Bao, H. *et al.* Hyperactivated Wnt-beta-catenin signaling in the absence of sFRP1 and sFRP5 disrupts trophoblast differentiation through repression of Ascl2. *BMC Biol.* **18**, 151 (2020). <https://doi.org:10.1186/s12915-020-00883-4>

6 Hemberger, M., Hughes, M. & Cross, J. C. Trophoblast stem cells differentiate in vitro into invasive trophoblast giant cells. *Dev. Biol.* **271**, 362-371 (2004). <https://doi.org:10.1016/j.ydbio.2004.03.040>

7 Zhu, D., Gong, X., Miao, L., Fang, J. & Zhang, J. Efficient Induction of Syncytiotrophoblast Layer II Cells from Trophoblast Stem Cells by Canonical Wnt Signaling Activation. *Stem Cell Reports* **9**, 2034-2049 (2017). <https://doi.org:10.1016/j.stemcr.2017.10.014>

8 Dai, J. *et al.* Chemoproteomics reveals baicalin activates hepatic CPT1 to ameliorate diet-induced obesity and hepatic steatosis. *Proc. Natl. Acad. Sci. U. S. A.* **115**, E5896-E5905 (2018). <https://doi.org:10.1073/pnas.1801745115>

9 Livak, K. J. & Schmittgen, T. D. Analysis of relative gene expression data using real-time quantitative PCR and the 2(-Delta Delta C(T)) Method. *Methods* **25**, 402-408 (2001). <https://doi.org:10.1006/meth.2001.1262>

**SUPPLEMENTARY FIGURES AND TABLES**

**
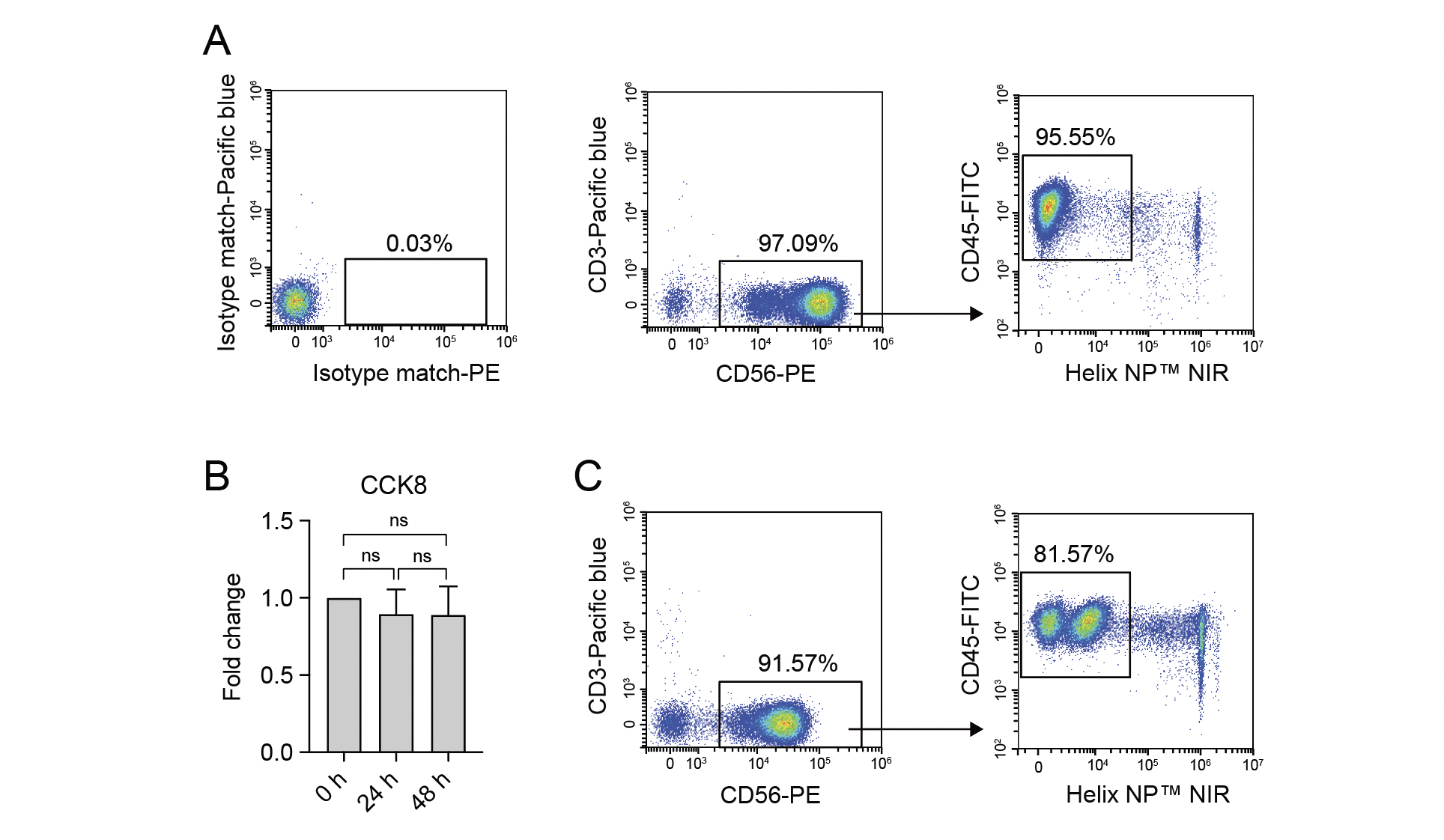
**

**Supplementary Figure 1. Purity and viability identification of isolated human dNK cells.** (A) Representative plots of flow cytometry showing the purity and viability of freshly isolated human dNK cells with Magnetic-activated cell sorting (MACS). (B) CCK8 assay showing the proliferative capacity of freshly isolated human dNK cells during *in vitro* culture for 48 hours. Data are based on three independent experiments and are presented as mean ± SEM. Statistical analyses were conducted using one-way ANOVA with Fisher’s LSD post-hoc test. ns, not significant. (C) Representative plots of flow cytometry showing the purity and viability of the frozen-thawed human dNK cells prior to adoptive transfer.

**
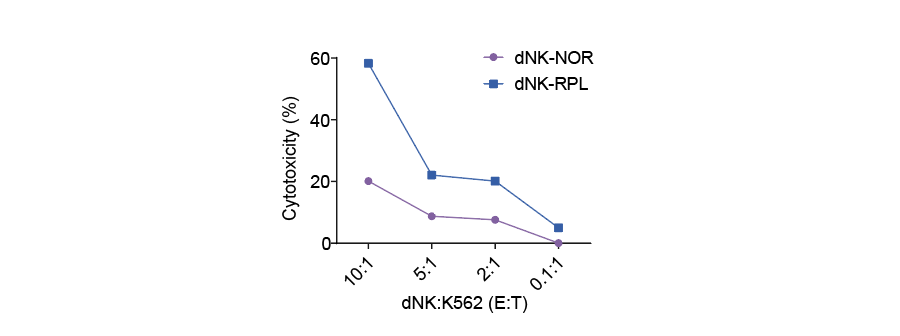
Supplementary Figure 2. Typical results showing cytotoxicity of human dNK cells from normal pregnant women (dNK-NOR) and RPL patients (dNK-RPL) against K562 leukemia cells.** E:T, effector cell: target cell.

**Supplementary Figure 3. The placental phenotypes in NOG mice that received adoptive transfer of human dNK cells from normal pregnant women and RPL patients.** (A) Representative images of immunohistochemical staining for cytokeratin 7 (CK7) in the placentae at E10.5 from Vehicle, NOR, and RPL groups. Pla, placenta; Dec, decidua. Scale bar, 100 μm (top row) and 20 μm (bottom row). (B-C) Statistical results revealing the number of invaded trophoblasts (B) and their maximum invasion distance (C) in decidual tissues from Vehicle, NOR, and RPL groups. (D) Quantitative real-time PCR analysis demonstrating marker gene expression related to trophoblast invasion in placentae at E10.5 from Vehicle, NOR, and RPL groups. (E) Representative images of H&E staining of the placentae at E10.5 from Vehicle, NOR, and RPL groups. Panels in the bottom row are a higher magnification of the indicated areas in the corresponding panels of the top row. Sp, spongiotrophoblast layer; Lab, labyrinth. Scale bar, 500 μm (top row) and 100 μm (bottom row). (E) Statistical results showing the ratio of labyrinth area to spongiotrophoblast layer area in the placentae at E10.5 from Vehicle, NOR, and RPL groups. Data were shown as mean ± SEM for panels B-D and F. Statistical analyses were performed by one-way ANOVA with Fisher’s post hoc test for panels B, C, and F and multiple *t*-test for panel D based on number of the placentae n=6 in Vehicle group, n=5 in NOR group, n=5 in RPL group. For B, C, and F, one or two views from each placenta were analyzed. *, p < 0.05; **, p < 0.01.

**Supplementary Figure 4.** **Gating strategy for the sorting of four dNK subsets.** Representative plots of flow cytometry showing the gating strategy for sorting the four dNK subsets collected from normal pregnant women at gestational week 6-9 (n=5). The CD16^-^CD56^+^ dNK cells were subset as CD39^-^CD103^-^ (cluster A), CD39^+^CD103^-^ (cluster B), CD39^+^CD103^+^ (cluster C), and CD39^-^CD103^+^ (cluster D).


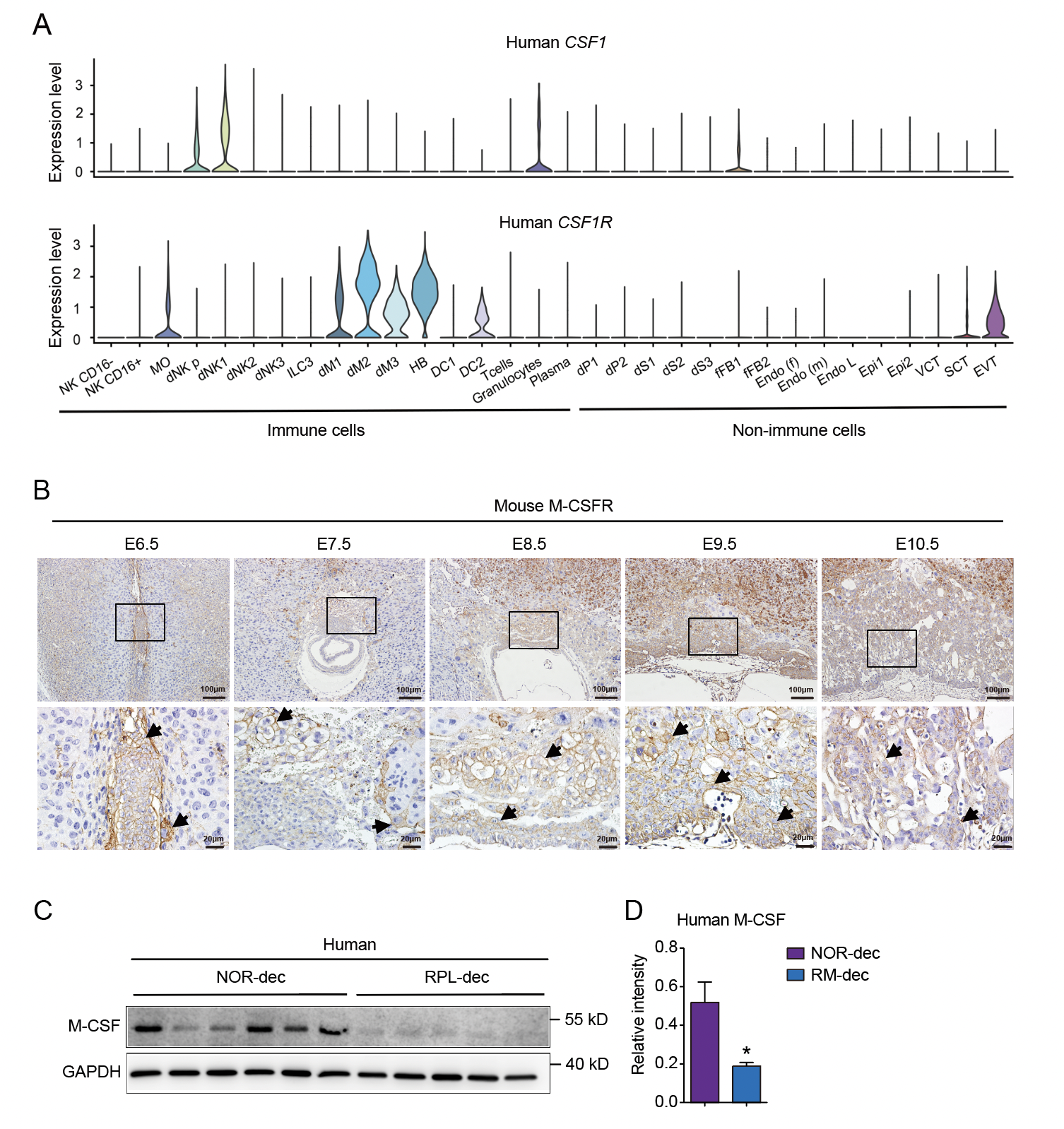


**Supplementary Figure 5.** **Expression pattern of M-CSF and M-SCF receptor at maternal-fetal interface.** (A) Violin plots based on scRNA-seq data (HRA000237 and GSE89497) showing the abundance of *CSF1* gene (encoding M-CSF) and *CSF1R* gene (encoding M-CSFR) in various cell subsets at human maternal-fetal interface during early pregnancy. NK, peripheral natural killer cells; MO, monocytes; dNK, decidual NK cells; dNKp, proliferative dNK cells; ILC, innate lymphoid cells; dM, decidual macrophages; HB, Hofbauer cells; DC, dendritic cells; dP, decidual perivascular cells; dS, decidual stromal cells; fFB, fetal fibroblast cells; Endo(f), fetal endothelial cells; Endo(m), maternal endothelial cells; Endo L, lymphatic endothelial cells; Epi, epithelial glandular cells; VCT, villous cytotrophoblast; SCT, syncytiotrophoblast; EVT, extravillous trophoblast. (B) Representative images of immunohistochemistry for M-CSF receptor (M-CSFR) at the maternal-fetal interface of C57BL/6 mouse from E6.5 to E10.5. The panels in bottom row are the magnifications of the rectangle regions in the corresponding panels in top row. The black arrows indicate M-CSFR^+^ trophoblast cells. Scale bars, 100 μm (top row), 20 μm (bottom row). (C-D) Western blotting analyses showing the protein level of M-CSF in the decidua of RPL patients (RPL-dec, n=5) and normal pregnancies (NOR-dec, n=6). GAPDH serves as internal control. (C) Representative blots. (D) Statistical analyses on the blots. The data in Panel D are presented as mean ± SEM and the statistical analysis was performed by Student’s *t*-test. *, p < 0.05.

**
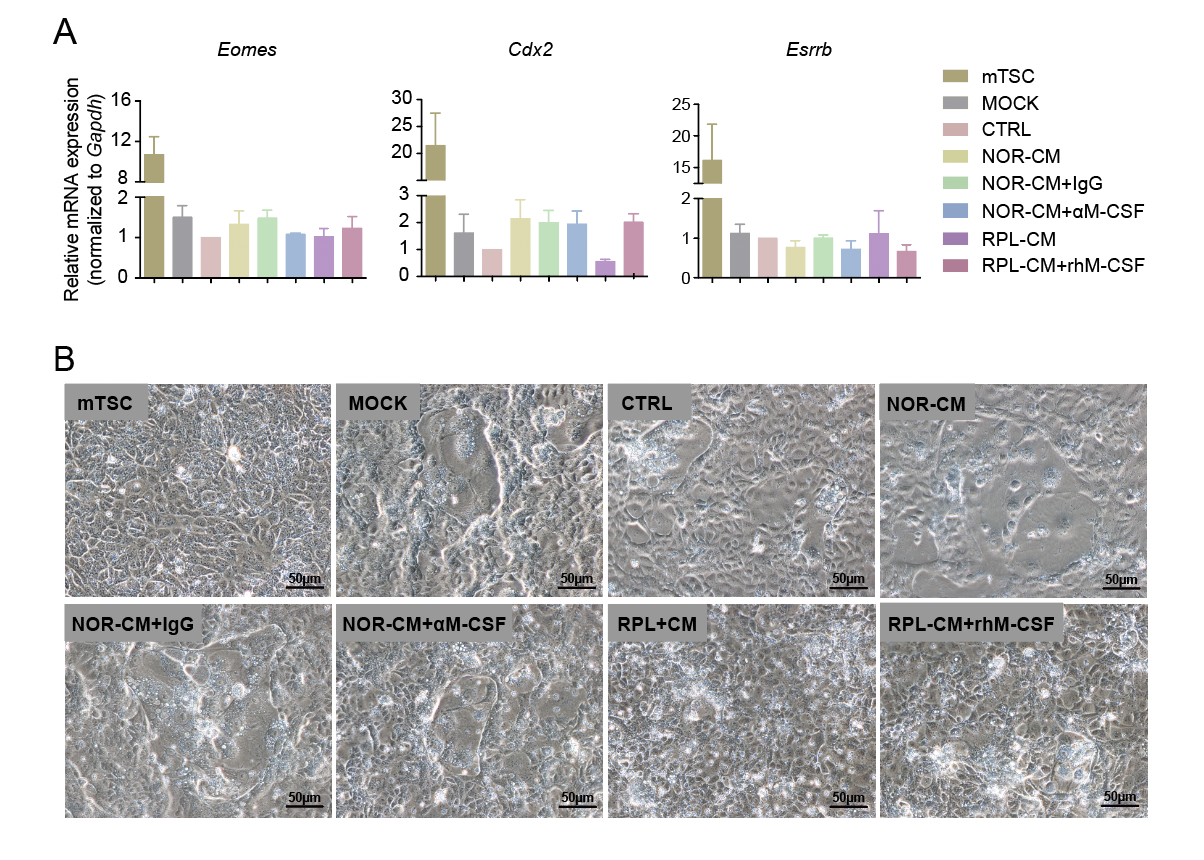
**

**Supplementary Figure 6. Effects of human dNK cells on mTSC differentiation *in vitro*.** (A) Statistical results of quantitative real-time PCR analysis for marker genes associated with stemness including *Eomes*, *Cdx2*, and *Esrrb* in mTSCs with various treatments shown as below. Data are presented as mean ± SEM based on the results from three independent experiments. (B) Morphological observation of mTSCs with various treatments shown as below. mTSC, mTSC in complete media. MOCK, mTSCs in differentiation media. CTRL, mTSCs in differentiation media supplemented with 50% dNK-free media. NOR-CM, mTSCs in differentiation media supplemented with 50% conditioned media of human dNK cells from normal pregnancies. NOR-CM-IgG, mTSCs in differentiation media supplemented with 50% conditioned media of human normal dNK cells that had been pre-incubated with pre-immune IgG. NOR-CM-αM-CSF, mTSCs in differentiation media supplemented with 50% conditioned media of human normal dNK cells that had been pre-incubated with neutralizing antibody against M-CSF. RPL-CM, mTSCs in differentiation media supplemented with 50% conditioned media of human dNK cells from RPL patients. RPL-CM-rhM-CSF, mTSCs in differentiation media supplemented with 50% conditioned media of RPL dNK cells and 1 ng/mL recombinant human M-CSF.

**
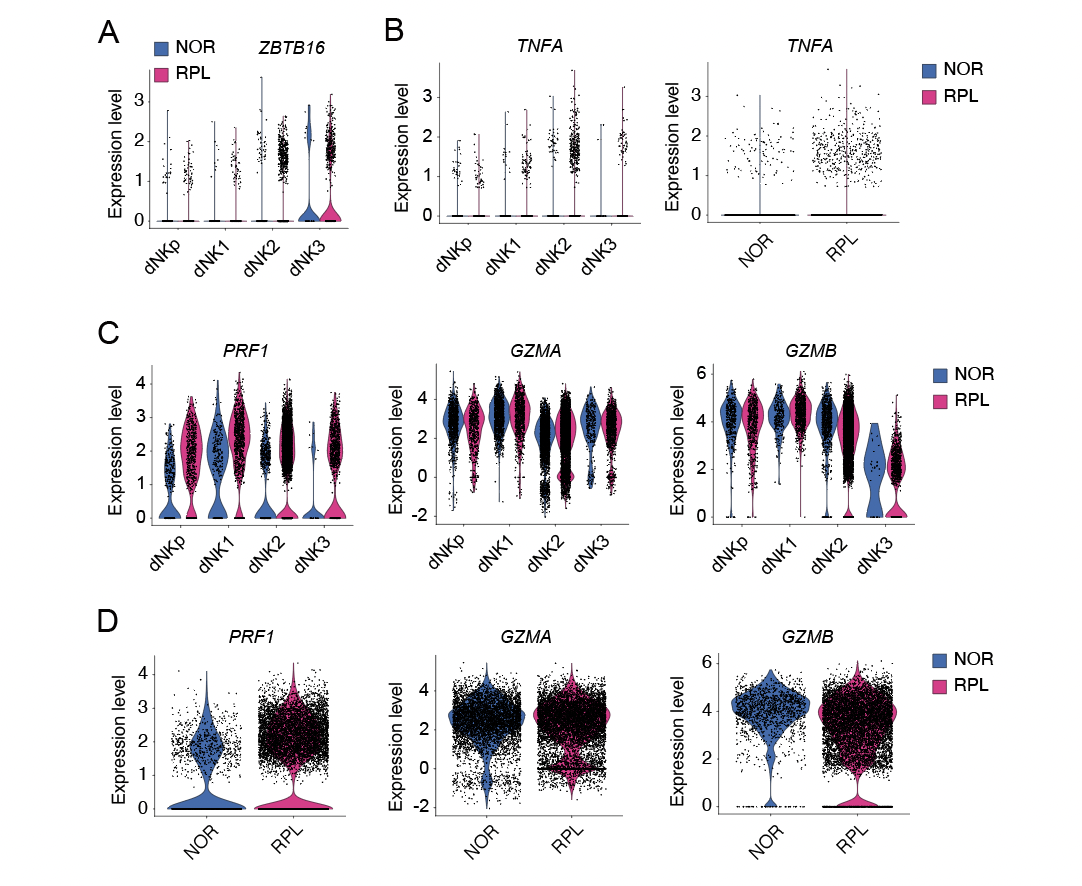
Supplementary Figure 7. Expression of inflammation-associated genes in human dNK cells from normal pregnant women (NOR) and RPL patients (RPL) in publicly scRNA-sequencing dataset.** (A) Violin plots based on scRNA-seq data (GSA: HRA000237) showing the relative expression of *ZBTB16* gene (a key regulator on *CD161*) in dNK subsets from RPL patients and normal pregnancy controls. (B) Violin plots based on scRNA-seq data (GSA: HRA000237) showing the relative expression of *TNFA* in dNK subsets or total dNK cells from RPL patients and normal pregnancy controls. (C-D) Violin plots based on scRNA-seq data (GSA: HRA000237) showing the relative expression of *PRF1, GZMA, and GZMB* in (C) dNK subsets or (D) total dNK cells from RPL patients and normal pregnancy controls.

Supplementary Table 1. Sequences of primers used for quantitative Real-time PCR.

| GENES | PRIMER SEQUENCE (5’~3’) | SOURCE |
| --- | --- | --- |
| Homo *GAPDH* | Forward: GAAGGTGAAGGTCGGAGTC | Invitrogen |
|  | Reverse: GAAGATGGTGATGGGATTTC | Invitrogen |
| Homo *HGF* | Forward: ACCCTGGTGTTTCACAAGCA | Invitrogen |
|  | Reverse: GCAAGAATTTGTGCCGGTGT | Invitrogen |
| Homo *IFNG* | Forward: CTGTCGCCAGCAGCTAAAAC | Invitrogen |
|  | Reverse: TACTGGGATGCTCTTCGACC | Invitrogen |
| Homo *TNFA* | Forward: CTCGAACCCCGAGTGACAAG | Invitrogen |
|  | Reverse: TATCTCTCAGCTCCACGCCA | Invitrogen |
| Homo *VEGFC* | Forward: ATGTGTGTCCGTCTACAGATGT | Invitrogen |
|  | Reverse: GGAAGTGTGATTGGCAAAACTGA | Invitrogen |
| Mus *Cdx2* | Forward: CCAGCTCTTTGCCTCTCTGT | Invitrogen |
|  | Reverse: TGCCTCTGGCTCCTGTAGTT | Invitrogen |
| Mus *Eomes* | Forward: AACATGCAGGGCAATAAGATG | Invitrogen |
|  | Reverse: AGCCTCGGTTGGTATTTGTG | Invitrogen |
| Mus *Esrrb* | Forward: GGGAGCTTGTGTTCCTCATC | Invitrogen |
|  | Reverse: CTACCAGGCGAGAGTGTTCC | Invitrogen |
| Mus *Gapdh* | Forward: GGAGAAACCTGCCAAGTATGATG | Invitrogen |
|  | Reverse: AAGAGTGGGAGTTGCTGTTGAAG | Invitrogen |
| Mus *Gcm1* | Forward: GCCTTACGAAGAGAAAGTATCTGTG | Invitrogen |
|  | Reverse: AGAACAGAAGTTTAGGAGCATCTCA | Invitrogen |
| Mus *Mct1* | Forward: TTGCCCCTTTGTCTACAACC | Invitrogen |
|  | Reverse: CAGCATTCCACAATGGTCAC | Invitrogen |
| Mus *Mct4* | Forward: GGATGGTCGTGCTTCATTTT | Invitrogen |
|  | Reverse: AATGGATCCAATCCAACCAA | Invitrogen |
| Mus *Prl2c2* | Forward: TGAGGAATGGTCGTTGCTTT | Invitrogen |
|  | Reverse: TCTCATGGGGCTTTTGTCTC | Invitrogen |
| Mus *Prl3b1* | Forward: CCAGAAAACAGCGAGCAAGT | Invitrogen |
|  | Reverse: AGGTACATGTGGAAGAGCAGC | Invitrogen |
| Mus *Prl3d1* | Forward: TGGTGTCAAGCCTACTCCTTT | Invitrogen |
|  | Reverse: CAGGGGAAGTGTTCTGTCTGT | Invitrogen |
| Mus *Syna* | Forward: TACCCTGTCTGTGGACACCA | Invitrogen |
|  | Reverse: ACCAGAGGAGTTGAGGCAGA | Invitrogen |
| Mus *Synb* | Forward: ATCCCCATAAGGACCGTTTC | Invitrogen |
|  | Reverse: AGGCAGAGAGGTTGCCTACA | Invitrogen |
| Mus *Tpbpa* | Forward: CGGAAGGCTCCAACATAGAA | Invitrogen |
|  | Reverse: TCAAATTCAGGGTCATCAACAA | Invitrogen |

Supplementary Table 2. List of primary antibodies used for flow cytometry and sorting.

| Antibodies | SOURCE | Catalog Number |
| --- | --- | --- |
| APC anti-human CD39 antibody | Biolegend | 328210 |
| APC anti-human CD49a antibody | Biolegend | 328314 |
| APC anti-human CD56 antibody | Biolegend | 318310 |
| FITC anti-human CD11b antibody | Biolegend | 301329 |
| FITC anti-human CD158 antibody | Biolegend | 339503 |
| FITC anti-human CD161 antibody | Biolegend | 339905 |
| FITC anti-human CD45 antibody | Biolegend | 368508 |
| FITC anti-human KIR2DL1/KIR2DS5 antibody | R&D Systems | FAB1844F-025 |
| Pacific Blue™ anti-human CD56 Antibody | Biolegend | 362519 |
| PE anti-human CD158d (KIR2DL4) antibody | Biolegend | 347005 |
| PE anti-human CD226 (DNAM-1) antibody | Biolegend | 338305 |
| PE anti-human CD27 antibody | Biolegend | 356405 |
| PE anti-human CD39 antibody | Biolegend | 328208 |
| PE anti-human CD56 antibody | Biolegend | 362508 |
| PE anti-human IFN-γ antibody | ebioscience | 12-7319-41 |
| PE anti-human IL-10 antibody | ebioscience | 12-7108-82 |
| PE anti-human IL-4 antibody | ebioscience | 12-7049-42 |
| PE anti-human TNF-α antibody | ebioscience | 12-7349-81 |
| PE anti-human KIR2DS1/CD158h antibody | R&D Systems | FAB8887P-025 |
| Pacific Blue anti-human CD3 antibody | Biolegend | 300330 |
| PerCP-Cyanine5.5 anti-human CD16 antibody | Biolegend | 302027 |
| PerCP-Cyanine5.5 anti-human CD3 antibody | ebioscience | 45-0037-42 |
